# Towards standardized microbial hydrogen consumption testing in the subsurface: harmonized field sampling and enrichment approaches

**DOI:** 10.1007/s11274-025-04542-0

**Published:** 2025-09-26

**Authors:** Kateřina Černá, Kristýna Fadrhonc, Jakub Říha, Petra Bombach, Sylvain Stephant, Caroline Michel, Laura Fablet, Joachim Tremosa, Kyle Mayers, Biwen Annie An-Stepec, Nicole Dopffel

**Affiliations:** 1 Institute for Nanomaterials, Advanced Technologies and Innovation, Technical University of Liberec, Studentská 1402/2, Liberec, 46117 Czechia; 2 Isodetect GmbH, Deutscher Platz 5b, Leipzig, 04103 Germany; 3 BRGM, 3 Avenue Claude Guillemin, Orléans Cedex 2, 45060 France; 4 Geostock, 2 Rue des Martinets, Rueil-Malmaison, 92500 France; 5 Norwegian Research Centre AS – NORCE, Nygårdsgaten 112, Bergen, 5008 Norway

**Keywords:** Biotic hydrogen loss, Microbial hydrogen consumption, Protocol standardization, Sulfate-reducing bacteria, Underground hydrogen storage

## Abstract

Hydrogen (H₂) will play a crucial role in Europe’s green energy transition, necessitating efficient storage solutions such as underground storage in salt caverns or porous media. However, the potential microbial H₂ consumption in these subsurface environments poses risks to storage stability and safety, and its magnitude remains relatively unexplored. Within the HyLife-CETP project, we developed a brine sampling protocol for the field operators and tested a standardized laboratory procedure for estimating microbial hydrogen consumption rates in these original brine samples, combining precise gas, chemical, and genetic analyses. Four labs tested and compared the developed enrichment protocol in a round-robin-like test using artificial brine and the hydrogen-consuming, sulfate-reducer *Oleidesulfovibrio alaskensis* as a reference strain. This test revealed consistent trends in microbial hydrogen consumption and corresponding pH increase across labs, indicating that the developed protocol effectively captures the overall microbial activity. However, inter-laboratory variability in the reported H_2_ consumption quantity, ranging from 19.8 to 61%, highlights how metabolic activity, analytical approaches, and sample handling variations can affect results. Most importantly, abiotic hydrogen loss, particularly leakage from experimental bottles, was identified as a significant factor affecting accurate hydrogen consumption estimation, and we tested different measures to reduce abiotic losses. Our developed protocol effectively detected microbial hydrogen consumption and identified associated metabolic processes, supporting its robustness for studying microbial activity in underground storage systems. In addition, the findings underscore the natural biological variability inherent to growth tests, particularly in hydrogen-utilizing systems.

**Supplementary Information:**

The online version contains supplementary material available at 10.1007/s11274-025-04542-0.

## Introduction

Hydrogen (H_2_) will play a vital role in the European green energy transition to balance the fluctuating supply of renewable energy generation and demand. Excess renewable electrical power (wind, solar) can be converted to H_2_ (Power-to-gas) and stored for later use. For such an energy system to be stable, sufficient storage volume is needed in approximately 30% of the expected energy demand (“HyUsPre-Storymaps,” 2022). Underground H_2_ storage in salt caverns or porous media-type storages like depleted gas fields or aquifers is a promising approach due to the available large-volume storage capacities, flexibility, and low operational costs (Beckingham and Winningham 2020; Tarkowski 2019). Further storage sites could be exploited by using offshore gas fields and leaching new salt caverns (Caglayan et al. 2020). The question remains whether all these potential sites are suitable for H_2_ storage. Introducing H_2_ into the subsurface can induce biotic and abiotic complex reactions, specifically microbial redox reactions. Many subsurface microorganisms can oxidize H_2_ coupled to the reduction of nitrate, ferric iron, sulfate, elemental sulfur, or carbon dioxide (CO_2_) to produce nitrogen, ferrous iron, hydrogen sulfide (H_2_S), acetate, or methane (CH_4_) (Gregory et al. 2019). Such microbial activity can induce a variety of microbial-triggered risks: (a) loss of the stored H_2_ and changes in gas composition, (b) risks to operational safety and deterioration in quality by H_2_S formation, (c) damage of the technical equipment by biocorrosion and microbial triggered precipitates, (d) subsequent environmental risks by H_2_S leakages outside the storage sites (e) changes of the reservoir properties by biofilm formation and precipitates (Dopffel et al. 2021).

Although microbial activity poses a significant risk to underground storage, microbial hydrogen (H₂) consumption in subsurface reservoirs remains poorly understood and appears to be highly site-specific, influenced by multiple factors such as geological setting, hydrogen availability, and electron acceptor presence (Dohrmann and Krüger 2023; Dopffel et al. 2023; Harris et al. 2007; Hellerschmied et al. 2024; Thaysen et al. 2021). Field studies have shown that microbial activity can significantly impact underground hydrogen storage (UHS), as demonstrated by incomplete H₂ recovery due to microbial methanogenesis in a depleted gas reservoir (Hellerschmied et al. 2024). Yet, broader assessments, such as the review by Thaysen et al. (Thaysen et al. 2021) concluded that only a minority of subsurface sites possess the geochemical conditions—such as suitable temperature, salinity, and pressure—to support key microbial processes, although only 5 out of 42 studied sites proved to be unsuitable for microbial growth. Recent modeling efforts focused on aquifers by Gao et al. (Gao et al. 2024) using coupled hydrological-mechanical-chemical-biological (HMCB) frameworks have further highlighted the complexity of interactions between geochemical parameters and microbial activity.

Safe, efficient, and economically viable hydrogen storage in underground reservoirs thus requires a thorough understanding of microbial activities. However, accurately assessing the microbial impact remains challenging due to the lack of standardized methodologies. Until now, there has been a significant variation in methods used to quantify microbial hydrogen consumption, which naturally impacts data comparability. The approaches can be categorized into two main groups: direct measurement techniques and indirect assessment methods. Direct measurement techniques are represented by the in-situ injection/withdrawal tests (Harris et al. 2007; Hellerschmied et al. 2024; Vítězová et al. 2023), or laboratory approaches such as a combination of pressure and gas composition monitoring (Dopffel et al. 2023; Khajooie et al. 2024; Ranchou-Peyruse et al. 2024) or experiments with a microfluidic system saturated with hydrogen (Liu et al. 2023). The indirect assessment includes the use of a tritium-based assay to estimate hydrogen oxidation in sediment samples (Adhikari et al. 2016). All these studies differ in total volume, gas-brine ratios, gas sampling techniques, applied materials/consumables, etc. Such methodological diversity complicates direct comparisons between studies. Therefore, implementing standardized protocols is essential to ensure consistency and reproducibility across laboratories, thereby minimizing variability caused by methodological differences.

Our study had two main objectives. First, we developed a protocol to quantify microbial hydrogen consumption using field-derived brine samples under controlled laboratory conditions. For this, we proposed a brine sampling protocol, which can be performed by field personnel at the site. The subsequent laboratory enrichment protocol was established through methodological alignment across four independent laboratories, integrating collective expertise and insights from relevant literature. Second, we evaluated the reproducibility and reliability of the protocol in an interlaboratory (round-robin) experiment using a mock brine sample. By comparing results across laboratories, we identified key steps contributing to variability and assessed the overall consistency of the method. This allowed us to verify the suitability of the protocol for future experimental studies and its potential for generating comparable data for modeling of microbial hydrogen consumption.

## Materials and methods

### Brine sampling at the field site

A brief overview of the sampling protocol is presented here; the full protocol, including a video tutorial on field brine sampling, is available in Online Resource 1. Water sampling was typically performed by two individuals: one managed the continuous flushing with nitrogen (N₂), while the other handled the brine collection. All procedures were conducted using sterilized gloves, and sampling materials were unsealed immediately prior to use to minimize the risk of contamination. Sterile, 1-liter pressure-resistant glass bottles were flushed with sterile N₂ gas to displace atmospheric oxygen. This was achieved using a sterile tube connected to an N₂ gas cylinder. Each bottle was flushed for a minimum of two minutes and maintained under a continuous N₂ flow until the sampling commenced. To ensure the collection of fresh brine, the system was allowed to run freely for 5–10 min to purge stagnant water. Following external disinfection of the sampling valve and tubing, a sterile sampling tube was attached, and the initial flow was discarded. Brine was then collected into the N₂-flushed bottles, which were filled to approximately 80% of their total volume. Throughout and after the collection, the bottle headspace was continuously flushed with N₂ to eliminate residual oxygen. The bottles were sealed with thick rubber stoppers and tightly capped. A total of 1.5–2 L of brine was collected for subsequent chemical analyses and microbial enrichments. In-situ reservoir temperature was recorded at the time of sampling. Following collection, the sample bottles were placed in styrofoam boxes and transported to the analytical laboratory as promptly as possible. Prior to enrichment, the brine was stored at room temperature in the dark to prevent salt precipitation, particularly in samples from salt caverns. When feasible, on-site filtration was performed using Millipore Sterivex Pressure Filter (Merck, Germany), filter size 0.22 μm, to preserve the original microbial DNA profile of the brine.

### Initial Brine analyses

Upon arrival at the analytical laboratory, brine samples were processed immediately to preserve their chemical and microbial integrity. All sampling procedures were conducted under anoxic conditions, either within an anaerobic glove box or by extracting samples through a surface-disinfected rubber stopper using a syringe and needle flushed five times with N₂ or Ar gas prior to use. The gas pressure and composition in the headspace were analyzed using gas chromatography (GC) to quantify hydrogen (H₂), hydrogen sulfide (H₂S), carbon dioxide (CO₂), and methane (CH₄). Additionally, oxygen (O₂) levels were monitored when technically feasible. Maintaining low oxygen levels throughout sampling, sample processing, and enrichment is crucial, as most anaerobic microorganisms tolerate oxygen concentrations only up to approximately 0.14%. (Lu and Imlay (Lu and Imlay 2021) and higher levels could compromise microbial viability and enrichment outcomes. High CO₂ concentrations were monitored as potential indicators of pH shifts in the original brine due to degassing. Chemical analyses were performed in accordance with the established protocols of each participating analytical laboratory. A summary of the recommended analyses, including sample handling and preservation protocols, is provided in Table1. These same parameters were also monitored during enrichment incubations.

**Table 1:** List of chemical analyses performed on Brine samples upon arrival

Method	Analytes	Purpose	Sample preservation
pH		pH change implies ongoing chemical/microbial reactions	Directly processed
Salinity (Lew et al. 2022)		Salinity is an important parameter for microbial growth and also for the setup of various chemical analyses	Directly processed
Eh (Whitfield 1969)		Redox state of the brine	Directly processed
Headspace pressure of the bottles		Pressure change implies ongoing chemical/microbial reactions	Directly processed
Gas composition (de Koning et al. 2009)	H_2_, O_2_, CO_2_, CH_4_, H_2_S	H_2_ consumption and production of other gasses are indicators of microbial activity.	Directly processed
Inorganic anions (Hodge et al. 2000)	Cl^−^, SO_4_^2−^, PO_4_^2−^	Overall chemical composition of the brine; changes in concentrations of these compounds (potential nutrients) might imply ongoing microbial activity	Sterile filter (0.22 μm) and store at room temperature (RT)
Dissolved HS^−^ (Cline and Richards 1969)		Changes in HS- concentrations imply the activity of SRB	For fluorometric measurements, the sample needs to be stabilized by 2 N zinc acetate (1 M Zn²⁺) solution (final concentration 2 mL per L).
Cations (Fernández-Turiel et al. 2000)	Na^+^, Ca^2+^, Mg^2+^, K^+^, NH_4_^+^, Fe(total)	Overall chemical composition of the brine	Acidify the sample with HNO3 to have pH < 2, store at RT
Organics compounds (Lawrence 1987)	Lactate, acetate + further organic acids and sugars	Overall chemical composition of the brine; changes in concentrations of these compounds (potential nutrients or metabolic products) might imply ongoing microbial activity	Directly or sterile filter (0.22 μm) and store it in the freezer
Total dissolved organic carbon (DOC) (Li et al. 2012)		Overall chemical composition of the brine; changes in concentrations of these compounds (potential nutrients) might imply ongoing microbial activity	Sterile filter (0.22 μm) and store it in the fridge
Total inorganic carbon (TIC)		Overall chemical composition of the brine; CO_2_/carbonates represent a crucial nutrient for methanogenesis	No filtration, directly processed (store in the fridge until analysis)

After chemical sampling, aliquots of 2 mL and 20 mL were collected in duplicate for genetic biodiversity analysis to establish a zero-point reference. These samples were centrifuged at 15,000 × g for 15 min, and the resulting pellets were stored at − 20 °C for subsequent DNA extraction and genetic profiling. These analyses were used to assess changes in microbial abundance and community composition during enrichment and to identify microbial groups involved in hydrogen consumption.

### Enrichment culture setup

On the first day of the experiment, eight sterile serum bottles (approximately 100 mL each; see Table 2) were prepared. The exact volume of each bottle (± 0.5 mL) was recorded, as this precision was essential for accurate quantification of hydrogen consumption. A minimum of eight samples was used to ensure reliable evaluation, although additional setups, such as nutrient-enriched brine or variable incubation temperatures, could be included to expand the experimental scope. All serum bottles were flushed thoroughly with nitrogen (N₂) to remove residual oxygen. Under anoxic conditions, six of the bottles (No. 1, 2, 3, 4, 7, and 8) were filled with exactly 50 mL of test brine, while bottles No. 5 and 6 were filled with 50 mL of sterile MilliQ water. Each bottle was sealed with a sterile, thick blue butyl stopper (19/13.2 × 12 mm) and secured with an aluminum crimp. New stoppers were used for each experiment and were pre-treated by boiling in distilled water three times to remove any wax, lubricant, or powder residues. In total, 400 mL of test brine was required for one complete experimental setup.

**Table 2:** List of enrichment culture samples to be set for each Brine sample

No	Liquid	Gas
1	Autoclaved brine sample	100% H_2_
2	Autoclaved brine sample	100% H_2_
3	Brine sample	100% H_2_
4	Brine sample	100% H_2_
5	Anoxic distilled H_2_O	100% H_2_
6	Anoxic distilled H_2_O	100% H_2_
7	Brine sample	100% N_2_
8	Brine sample	100% N_2_

Bottles No. 1 and 2 were sterilized by autoclaving at 121 °C and > 205 kPa for 20 min on three consecutive days, with 24-hour incubation at room temperature between each cycle. The remaining bottles were stored at room temperature in the dark during this period. Following sterilization, the headspace of bottles No. 1–6 was flushed with 100% hydrogen gas for approximately one minute, and the internal pressure was adjusted to ~ 1.75 bar (0.75 bar overpressure). Bottles No. 7 and 8 were flushed with 100% N₂ and similarly pressurized. All bottles were incubated upside down at reservoir temperature for at least one hour before initial gas composition analysis by gas chromatography (GC). A 0.3 mL aliquot was withdrawn from each bottle using an N₂-flushed syringe for pH measurement (time point zero). After an additional hour of incubation, headspace pressure was measured again to establish baseline values. Throughout the experiment, samples were incubated upside down to minimize hydrogen loss through the butyl stoppers. Incubation was conducted in the dark, without agitation, and at a constant temperature corresponding to the reservoir conditions. The duration of the experiment was sufficient to allow for at least four sampling points beyond the initial time point. At each sampling point, anoxic sampling was performed directly from the enrichment bottles using an N₂-flushed syringe. Headspace pressure was measured first, followed by GC analysis of H₂, CO₂, CH₄, and H₂S. If the pressure dropped to atmospheric levels or below the threshold for reliable GC analysis, the bottles were re-pressurized by injecting 10 mL of 100% N₂. This step was critical to avoid underpressure, which increases the risk of oxygen contamination. Following GC analysis, a 0.3 mL brine sample was collected for pH measurement. Bottles were then incubated for at least one additional hour before a final pressure reading was taken. Omitting this final measurement could result in overestimation of hydrogen consumption. At least once during the experiment, additional brine was collected for organic acid and DNA analyses. A 2 mL sample was centrifuged at 15,000 × g for 15 min; the supernatant was used for chemical analysis, and the pellet was stored at − 20 °C for DNA extraction. At the final sampling point, the same procedures were followed. Additionally, a 20 mL brine sample was collected, centrifuged at 15,000 × g for 15 min, and the pellet was frozen at − 20 °C for DNA extraction (to be used if the DNA yield from the 2 mL sample was insufficient). The supernatant was used for chemical analysis as outlined in Table 1. Due to limited sample volume, the final analyses focused on compounds likely to serve as nutrients or metabolic byproducts, enabling comparison with the original brine and supporting the identification of active microbial processes during enrichment.

### Microbial hydrogen consumption calculation

To calculate the amount of H_2_ in the bottles, the ideal gas law (1) is used to correlate temperature (T in Kelvin), and pressure (p in Pascal) in the bottles minus water vapor pressure at the given temperature (in Pascal), and the gas constant (8.3144 J⋅K^−1^⋅mol^−1^). The measured composition of the gas phase (%) and the known volume of the bottle (118 mL – liquid medium + additions) is used to calculate the gas volume of H_2_ in the bottles containing the medium V (in m^3^):

p x V = n x R x T (1).

For each sample, the volume of H_2_ before and after the sampling procedure was calculated by measuring pressure at the beginning and the end of the sampling. The loss of H_2_ in between is related to the withdrawal of gaseous and liquid samples. This loss through sampling can be calculated and subtracted from the first pressure value calculated to obtain the volume of consumed H_2_. Additionally, abiotic H₂ loss resulting from system leakage must be accounted for based on the distilled water control samples incubated with hydrogen (Table 2) to calculate net microbial consumption. Absolute maximum net rates are obtained by calculating the slope of H_2_ consumption during days. Relative net rates are computed by normalizing the H_2_ values to the initial starting value, resulting in %-loss over time.

### Round-robin test

Based on the protocol described above, an inter-laboratory comparison was conducted using artificial brine enriched with a single microbial strain to assess the protocol’s reliability and reproducibility. Four laboratories (denoted as Laboratories 1–4 for anonymization) participated in this evaluation.

### Artificial brine sample preparation for round-robin test

Laboratory 3 prepared and distributed a 50 mL culture of the sulfate-reducing bacterium *Oleidesulfovibrio alaskensis* G20 (DSM 17464; Feio et al. 2004; Waite et al. 2020), which uses H₂ as an electron donor. In addition, approximately 1 L of anoxic culture medium suitable for this strain was provided by Lab 3 to the participating laboratories. The culture media for*Oleidesulfovibrio alaskensis* was prepared based on the prescription for medium 195c (Koblitz et al. 2023) with some modifications, the composition of which is summarized in Table3. Solution A was set up with 950 mL of water and autoclaved in a Widdel flask. While hot, it was taken out and flushed with a gentle stream of 80% N_2_ and 20% CO_2_ gas mixture until cooled down. Solutions B and E were autoclaved under a 100% N_2_ gas atmosphere. Solution D was prepared under a 100% N_2_ gas atmosphere and sterilized by filtration. After the mixture A had cooled down, 50 mL of solution C was added. Then, solutions B, D, and E were added. We used 1.25 mL of a 1 M stock solution E. The end media volume was 1012.25 mL (Table 3). The final media pH should be 7-7.4 and was adjusted by adding anoxic and sterile HCL (1 M) or NaOH (1 M) if needed.

**Table 3:** Composition of the culture medium for Oleidesulfovibrio alaskensis (amended medium 195c from DSMZ; Koblitz et al. 2023)

Solution A	
Na_2_SO_4_	3 g
KH_2_PO_4_	0.2 g
NH_4_Cl	0.3 g
NaCl	21 g
MgCl_2_ × 6H_2_O	3 g
KCl	0.5 g
CaCl_2_ × 2H_2_O	0.15 g
Sodium acetate	0.8203 g
Distilled water	950 mL
Solution B	
Trace element solution SL-10 (medium 320)	1 mL
Solution C	
Na_2_CO_3_	1.5 g
Distilled water	50 mL
Solution D	
1x Wolin’s vitamin solution	10 mL
Solution E	
1 M solution of Na_2_S x 9H_2_O	1250 µL
**Total volume**	**1012.25 mL**

### Round-robin protocol

Each laboratory began the experiment within the same period, with a maximum difference of one month. The experiment was initiated by transferring 2.5 mL of the original *Oleidesulfovibrio alaskensis* culture into 50 mL of fresh anoxic culture medium containing 20 mM sodium lactate under strictly anoxic conditions. The culture was incubated at 37 °C for 72 h until it became visibly turbid, serving as a pre-culture. From the 1 L provided *Oleidesulfovibrio alaskensis* sterile culture medium, exactly 50 mL of medium was dispensed into two sterile 100 mL serum bottles, previously flushed with N₂. These were designated as sterile brine samples No. 1 and No. 2 (see Table 2). The entire 50 mL of pre-culture was then transferred into the remaining ~ 900 mL of culture medium using an N₂-flushed syringe, creating our test brine. Since a fast-growing single bacterial strain was used, only 1 mL of sample was collected for genetic analysis at each sampling point.

### Round-robin chemical analytics

Significant attention was paid to methodical alignment during the round-robin test planning. However, due to technical limitations (available instrumentation, methods standardly used), it was decided to standardize only the list of target analytes. Each laboratory was allowed to select analytical methods based on its own experience and expertise. We focused on key analyses essential for the estimation of microbial consumption rates: gas composition via GC, pH, the concentrations of SO₄²⁻ via the barium sulfate assay (Houot and Duhamet 1992) or IC (Cole and Evrovski 1997) analysis, aqueous HS⁻ via the Cline assay (Cline and Richards 1969), and acetate via HPLC (Lawrence 1987). The specific analytical methods used by each laboratory to quantify these compounds are summarized in Online Resource2. The acetate data were collected regularly during the experiment in all the labs except Lab 4, where only the beginning and end-point data were available. Sulfate concentrations were quantified at the end of the experiment and compared to the initial brine values in all laboratories. Sulfate loss was converted into theoretical hydrogen consumption using sulfate reduction stoichiometry and the ideal gas law.

The theoretical chemical composition of synthetic brine was calculated from the prescription for the media preparation using the geochemical code PHREEQC (Parkhurst and Appelo 2013) and the Thermoddem database (Blanc et al. 2012), designed for environmental studies in natural geochemical systems. Water/gas exchange and redox speciation were considered in the calculation. Values measured by each lab were compared to these expected values.

### Round-robin genetic analyses

The pellets from 1 mL samples taken during the experiment were used for DNA extraction with the DNeasy^®^ Blood & Tissue Kit (Qiagen, Germany). Initially, 20 µL of Proteinase K and 180 µL of buffer ATL were added directly to the stored pellet and incubated at 56 °C for 15 min or until fully lysed. Subsequently, we followed the manufacturer’s protocol, using only 50 µL of final extraction buffer AE in the last step. The DNA concentration was measured fluorometrically using a Qubit^®^ 2.0 Fluorometer (Invitrogen, Life Technologies) following the manufacturer’s protocol. The extracted DNA was stored in the freezer (−20 °C) until further analyses. The extracted DNA was used to estimate 16 S rRNA gene copy numbers as a measure of microbial abundance in the samples. The digital droplet PCR (ddPCR) or digital PCR (dPCR) was performed by Lab 3 and Lab 4, respectively, using the same universal bacterial primers Prba338f (5´-ACTCCTACGGGAGGCAGCAG-3’) and Prun518r (5´-ATTACCGCGGCTGCTGG-3’) (Ovreås et al. 1997) for V3 region of 16 S rRNA gene.

At Lab 3, ddPCR reactions were run with a total volume of 20 µL on a DX200 instrument (Bio-Rad Laboratories, USA) using 5 µL of template DNA, 1 x EvaGreen supermix (Bio-Rad Laboratories, USA), and 250 nM (final concentration) of primers and ultra-pure water. Complete PCR reactions were emulsified with QX200 Droplet Generation Oil for EvaGreen using the QX200 Droplet Generator, then transferred to a ddPCR 96-well plate. The PCR reaction was performed using these plates in a C1000 Touch thermocycler with deep-well module (Bio-Rad Laboratories, USA) using the following settings: 95 °C for 5 min, 40 cycles of 95 °C for 30 s then 61 °C (bacteria) or 57.1 °C (SRBs) for 1 min, 4 °C for 5 min, 90 °C for 10 min and finally an infinite hold at 4 °C. Plates were equilibrated to room temperature for at least 10 min before analysis using the QX200 Droplet Reader (Bio-Rad Laboratories, USA). To distinguish between positive and negative droplets, we manually set a threshold for cut-off based on the positive (bacteria cultures) and negative (ultra-pure water) controls.

The dPCR analysis at Lab 4 was performed using the QIAcuity system (Qiagen, USA) with the use of fluorescent dye EVA Green. dPCR mixtures with a total volume of 12 µl were prepared using 3x EvaGreen PCR Master Mix (Qiagen, USA), 4.8 pmol of each primer (Generi Biotech, Czech Republic), and 1 µl of template DNA. Each sample was analyzed in duplicate in a QIAcuity Nanoplate 8.5 K 24-well plate (Qiagen, Germany) together with a blank control (no template control - NTC). The dPCR temperature conditions were as follows: initial denaturation at 95 °C for 2 min, followed by 40 cycles (30 at 16 S) (95 °C for 15 s, 60 °C for 15 s, 72 °C for 15 s) and 40 °C for 5 min. The amplification was followed by image acquisition (imaging) for 200 ms, gain 2.

The obtained 16 S rRNA gene copy numbers were recalculated for the number of microbial cells in the sample per 1 L of the brine, considering that the *Oleidesulfovibrio alaskensis* strain has four 16 S rRNA gene copies in its genome (Hauser et al. 2011).

### Additional tests on abiotic hydrogen loss

Two additional tests were conducted because we observed differences in both overall microbial hydrogen consumption rates and abiotic hydrogen loss. Lab 1 and 3, observing the largest abiotic H_2_ loss, optimized their setups and performed a new test on abiotic loss. Two 100 mL sterile serum bottles were filled with 50 mL distilled water, sealed with new sterile thick (19/13.2 × 12 mm) blue butyl stoppers, appropriately washed to remove all the surface preservatives, and crimped tightly with solid aluminum crimps. The headspace was flushed for 1 min with 100% H₂, and the headspace pressure was set to ~ 0.7 bar overpressure (1.7 bar absolute). The samples were incubated at 37 °C for at least 1 h; then, the headspace pressure was measured, followed by the GC measurement. The bottles were then incubated again for 30 min at 37 °C, and the pressure was remeasured. The pressure and gas composition measurement was repeated on days 2, 4, 7, and 9. The H₂ loss was estimated as described above and compared between the two labs.

The second test, denoted as the simulated hydrogen consumption test, was performed by all four labs and targeted the precision of hydrogen decrease detection. Two samples filled with distilled water were prepared similarly to the previous test. The pressure and gas composition were again measured on days 0, 2, 4, 7, and 9. Contrary to the previous test, 5 mL of gas was removed using a 5 mL plastic syringe and needle before each measurement (starting on day 2) to simulate the H_2_ consumption. The bottle with reduced gas content was again warmed up for 30 min to 37 °C, and pressure measurement and GC analysis were performed. Finally, the sample was heated again, and a final pressure measurement was performed. The H₂ decrease was estimated and compared between labs.

#### Statistical analysis

For each lab and analyte, we calculated the mean value, standard deviation, and coefficient of variance across the labs and the differences from expected values (in %). The correlation between the overall net hydrogen consumption (in %) and pH change compared to the zero-point was estimated using non-parametric Spearman’s rank correlation rho.

## Results

In the evaluation, we focused on three different aspects: (1) chemical analytics of the original brine, (2) hydrogen consumption rates, and (3) additional parameters measured in enrichment cultures necessary for the description and interpretation of microbial and chemical changes occurring in our experimental system.

### Brine chemical analytics

Each laboratory performed brine chemical analytics (Online Resource 2), and the results are summarized in Table 4. The PHREEQC geochemical calculation code was used to calculate the theoretical composition. The calculated brine composition and the difference from the expected value (DFE) were estimated for each analyte and lab in Table 4. We also compared the measured values regardless of the predicted value; these statistics are also given in Table 4.

**Table 4:** Brine composition detected by different laboratories and the theoretical expected values (Exp.) calculated by PHREEQC based on media composition. DFE - difference from expected value in %; Mean - mean value of measured values; SD - standard deviation of measured values; CV - coefficient of variation of measured values

Analyte	Exp.	Lab 1	DFE	Lab 2	DFE	Lab 3	DFE	Lab 4	DFE	Mean	SD	CV
pH	**7.32**	7.39	1.0%	7.45	1.8%	7.05	−3.7%	6.99	−4.5%	**7.2**	**0.2**	**2.8%**
SO_4_^2−^ (mg/L)	**1852**	1741	−6.0%	1700	−8.2%	1887	1.9%	1760	−5.0%	**1772**	**69.8**	**3.9%**
Cl^−^ (mg/L)	**14,692**	13,844	−5.8%	14,000	−4.7%	14,200	−3.3%	14,300	−2.7%	**14,086**	**176.6**	**1.3%**
Sulfide (aqueous) (mg/L)	**41**	12.8	−68.8%	21	−48.8%	27.16	−33.8%	25.4	−38.0%	**21.6**	**5.5**	**25.7%**
Acetate (mg/L)	**653.3**	593.9	−9.1%	650.5	−0.4%	664.8	1.8%	652	−1.0%	**639**	**27.4**	**4.2%**

The variability among the laboratories and differences from the expected values (DFE) were below 10% (and mostly below 5%) for all the compounds in all the laboratories except for the aqueous HS^−^ measurement, which resulted in considerable variability. Furthermore, the estimated DFE values of individual analytes were comparable to the estimated coefficients of variation (CV) of measured values, implying good reproducibility of the results among the laboratories. On the other hand, high DFE and CV values of aqueous HS^−^ concentrations demonstrated that reliable analysis of unstable aqueous HS^−^ concentrations is a highly complex task and needs further methodical improvement across laboratories.

### Hydrogen consumption rates

**Fig. 1:**
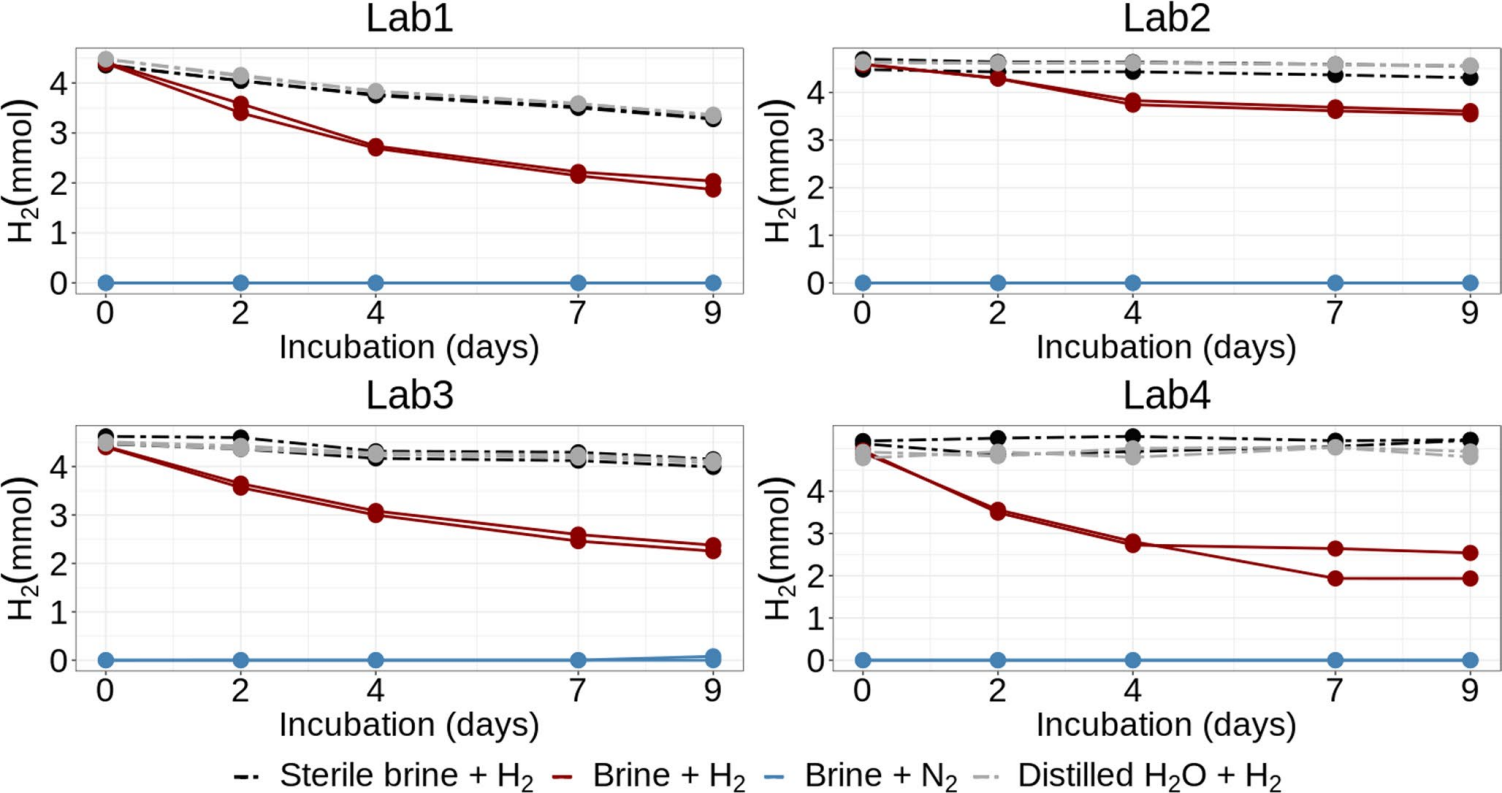
H_2_ consumption (in mmol) in the headspace during the experiment

We first evaluated the hydrogen consumption (expressed in mmol) during the experiment, which was corrected for the sampling loss using the computation approach. The original data are presented in Online Resource 3. All the labs observed no lag phase in the hydrogen consumption and a considerable decline in hydrogen molar content compared to the sterile controls (Fig. 1). However, we observed variability among the labs both in microbial H_2_ consumption rates (as observed in brine samples incubated with H_2_) and abiotic H_2_ loss (as detected in distilled water and sterile brine control samples incubated with H_2_), Fig. 1.

In Lab 1, the hydrogen content in the living brine samples decreased from an average of 4.4 mmol to 2.0 mmol, corresponding to a mean loss of 55%. In the sterile brine samples, the hydrogen content declined to an average of 3.3 mmol, representing a mean loss of 25%. In Lab 2, the hydrogen content in the living samples dropped from an average of 4.6 mmol to 3.6 mmol (a mean loss of 22%), while in the sterile samples, it remained relatively stable, decreasing only to 4.4 mmol (a mean loss of 3.5%). In Lab 3, the hydrogen content in the living brine samples decreased from an average of 4.4 mmol to 2.3 mmol (a mean loss of 47.5%), whereas in the sterile samples, it dropped slightly to 4.1 mmol (a mean loss of 10%). Finally, in Lab 4, the hydrogen content in the living samples declined from an average of 4.9 mmol to 2.2 mmol (a mean loss of 55%), while in the sterile samples, it remained stable (Fig. 1).

Because H₂ is a highly diffusive and permeable molecule, it tends to leak from experimental systems gradually. This can lead to an overestimation of overall microbial hydrogen consumption. To account for this, we also measured abiotic hydrogen loss from distilled water samples to assess the integrity of the experimental setup. After 9 days, no (or negligible) hydrogen loss was detected in Labs 2 and 4. In contrast, Lab 1 exhibited a significant hydrogen loss of approximately 25% due to leakage, while Lab 3 showed a loss of 8.8% over the same period (Online Resource 4, Figure S1).

**Fig. 2:**
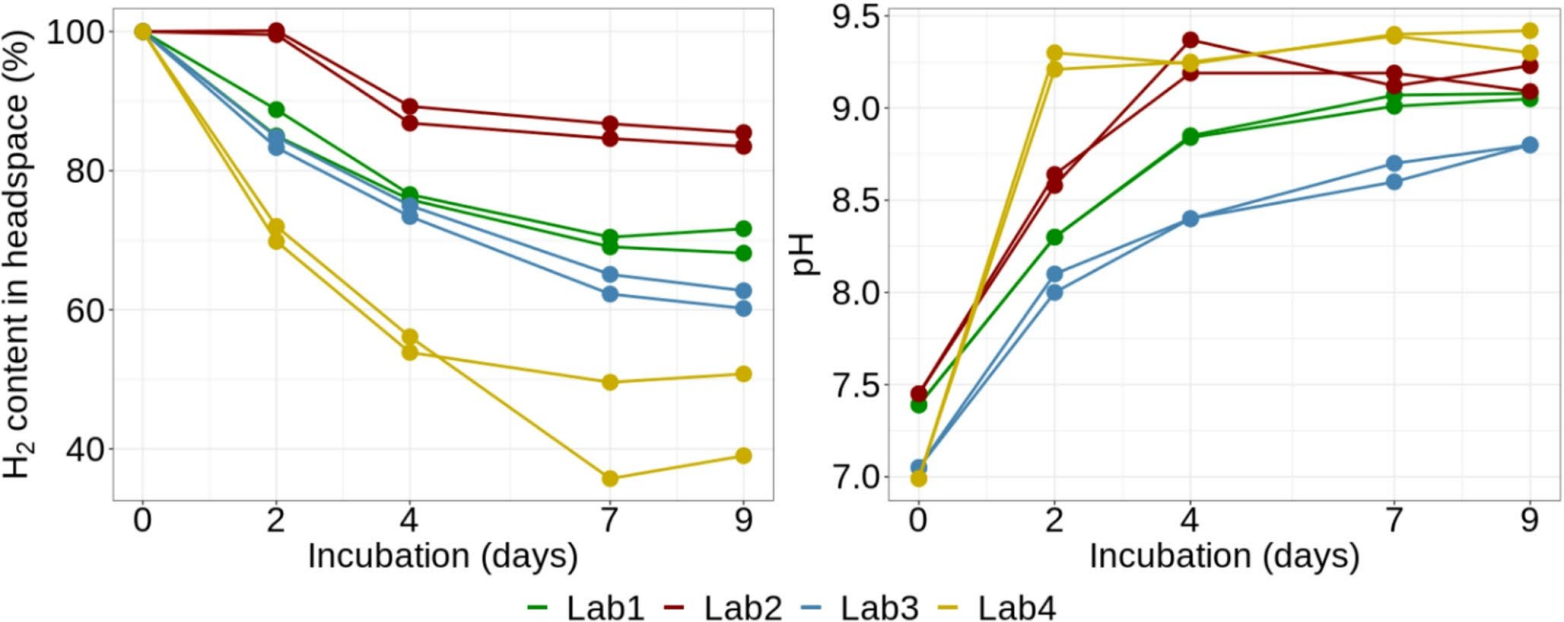
Left: The net microbial H_2_ consumption (in %) in brine samples incubated with hydrogen, corrected for the sampling loss and abiotic hydrogen loss during the experiment, detected in each lab. Right: Evolution of pH during the experiment in the brine samples incubated with hydrogen

Because of the observed relatively high interlaboratory variability in abiotic hydrogen loss, the net microbial hydrogen consumption (in mmol and in %) in living brine samples incubated with hydrogen was calculated by subtracting hydrogen loss attributable to sampling and abiotic loss. This approach allowed for a more accurate estimation of microbial hydrogen consumption and enabled better comparison between laboratories. Lab 4 observed the highest net hydrogen consumption in the brine samples, with 49.2–61% (2.4 and 3 mmol, respectively) of the initial hydrogen consumed by microbial activity over 9 days. In contrast, Lab 2 showed the lowest consumption, ranging from 19.8 to 21.5% (0.9 and 1 mmol, respectively). Lab 1 reported a net hydrogen consumption of 28.3–31.9% (1.3 and 1.4 mmol, respectively), while Lab 3 ranged from 37.3 to 39.8% (1.6 and 1.8 mmol, respectively) (Fig. 2). The maximum net hydrogen consumption rates observed were − 0.75, − 0.33, − 0.27, and − 0.37 mmol/day for Labs 1, 2, 3, and 4, respectively. In all cases, these peak rates occurred during the initial phase of the experiment, between day 0 and day 2.

Furthermore, the pH increase proved a good indicator of ongoing microbial hydrogen consumption for sulfate reducers. The pH increase was positively correlated with the H_2_ percentage loss across the samples in our experiment (Spearman’s rank correlation test, *p* = 3.942e-14). The more prominent pH changes in sterile brine samples incubated with H_2_ or brines incubated with N_2_ were observed only at the beginning of the experiment in most labs, when CO_2_ degassing probably occurred due to brine manipulation and gas exchanges during the enrichment setup. A much more prominent and consistent increase in pH was observed in brine samples incubated with hydrogen, where the pH increased from the original pH values of 6.9–7.7 up to 8.7–9.4 (Fig. 2). The highest pH (9.3 and 9.4) was observed in the case of Lab 4, where the highest microbial hydrogen consumption rate was also detected (Fig. 2).

### dPCR/ddPCR

**Fig. 3:**
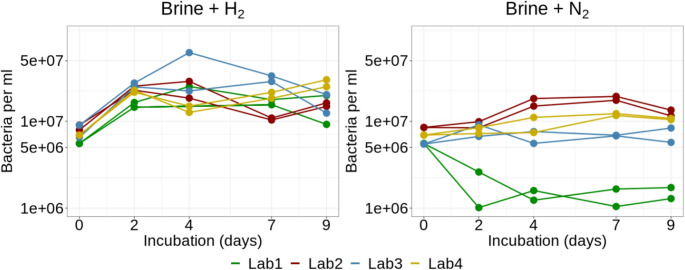
Biomass quantification in the brine samples by (droplet) digital PCR

Two independent labs (Lab 3 and Lab 4) performed dPCR/ddPCR analyses using the same primers following the protocol described in the method section. In the original brine samples, an average of 7,371,259 cells/mL (SD = 1460757) was detected in all four labs based on digital PCR analysis. A consistent signal was also detected in the brine samples incubated with hydrogen across all four labs, showing a moderate increase in microbial abundance throughout the experiment. Notably, approximately 2–3 × 10⁷ cells/mL were detected in the sample collected on the second day, and the microbial abundance remained relatively stable for the rest of the experiment (Fig. 3).

Lower cell counts and higher variability in the cell numbers among the labs were detected in the case of brine samples incubated with nitrogen. The detected cell numbers were approximately 10^7^ cells/mL in Labs 2, 3, and 4, which is nearly the same density as in the original brine samples. The observed cell density was stable across the experiment. However, a decrease in microbial abundance was observed in the Lab 1 brine samples incubated with nitrogen at the beginning of the experiment, with only 10⁶ cells/mL detected from the second day onward (Fig. 3).

### Nutrient consumption

We monitored acetate and sulfate concentrations as the primary nutrients in our experimental brine system together with the production of gaseous hydrogen sulfide (aqueous sulfide could not be analyzed due to limited sample volume).

Acetate levels remained stable in brine samples incubated under a hydrogen atmosphere across all laboratories, as well as in all nitrogen-incubated setups—except for Lab 1, where analytical issues affected the measurements (Online Resource 4, Figure S2). These results indicate that the tested strain utilizes only minimal amounts of acetate as a carbon source.

Sulfate concentrations remained consistent throughout the experiment in the sterile brine samples and in those incubated with nitrogen across all laboratories (Table 5), suggesting that no sulfate reduction occurred in these conditions. In contrast, a significant decrease in sulfate concentration was observed in brine samples incubated with hydrogen in all laboratories (Table 5), providing clear evidence of ongoing microbial sulfate reduction.

**Table 5:** Concentration of sulfate (mg/L) detected in brine samples in the four labs. The declared analytical precision of each lab is indicated in the lab names

		Lab 1 (± 10%)		Lab 2 (± 2%)		Lab 3 (± 10%)		Lab 4 (± 10%)	
	Theoretical entration	Start	9 days	Start	9 days	Start	14 days	Start	9 days
Sterile brine	1852	1741	1736	1700	2000	1887	2000	1760	1860
Sterile brine			1814		1900		1813		1870
Brine + H_2_			1309		950		907		722
Brine + H_2_			1314		910		1013		772
Brine + N_2_			1896		1900		1840		1814
Brine + N_2_			1845		1900		1893		1830

Measurements of hydrogen sulfide in the gas phase revealed very low concentrations, with the compound undetected in most analyses conducted by Lab 1, Lab 3, and Lab 4 (Online Resource 4, Figure S3). This pattern reflects the dissolution behavior of H_2_S, which is completely retained in the aqueous phase at pH ≥ 8 (Millero 1986). Accordingly, in our active brine samples with elevated pH (Fig.2), most of the produced H₂S remained in the liquid phase.

The detected sulfate loss was recalculated into theoretical hydrogen consumption using sulfate reduction stoichiometry and the ideal gas law. Due to significant variability in the initial sulfate concentrations measured by individual laboratories—and the frequent observation that final sulfate values exceeded initial ones—theoretical sulfate concentrations based on the defined media composition were also incorporated into the calculations.

Overall, the net hydrogen consumption based on gas analytics data, corrected for sampling and abiotic losses, aligned well with theoretical estimates derived from sulfate consumption, particularly when theoretical initial sulfate concentrations were used (Table 6). In Lab 1, the net hydrogen consumption (1.25 mmol and 1.40 mmol) slightly exceeded estimates based on both measured (0.90 mmol and 0.89 mmol) and theoretical (1.13 mmol and 1.12 mmol) sulfate consumption. A similar pattern was observed in Lab 4, where net hydrogen consumptions (2.44 mmol and 2.99 mmol) were also slightly higher than the corresponding estimated based on the measured (2.16 mmol and 2.06 mmol) and theoretical (2.35 mmol and 2.25 mmol) sulfate values. These findings suggest a mild overestimation of hydrogen consumption in Labs 1 and 4.

**Table 6:** Hydrogen consumption (in mmol) during the experiment - calculated value based on sulfate consumption considering measured zero-point sulfate concentrations (CALreal) or theoretical zero-point sulfate concentration based on media composition (CALtheor) together with the net hydrogen consumption (mmol) estimated in our samples based on gas analytics

	Lab1			Lab2			Lab3			Lab4		
	CAL_real_	CAL_theor_	Net H_2_ consumption	CAL_real_	CAL_theor_	Net H_2_ consumption	CAL_real_	CAL_theor_	Net H_2_ consumption	CAL_real_	CAL_theor_	Net H_2_ consumption
Brine + H_2_	0.90	1.13	1.25	1.56	1.88	0.91	2.04	1.97	1.85	2.16	2.35	2.44
Brine + H_2_	0.89	1.12	1.40	1.65	1.96	0.99	1.82	1.75	1.88	2.06	2.25	2.99

In contrast, Lab 3 showed strong agreement between net hydrogen consumption (1.85 mmol and 1.88 mmol) and both sulfate-based estimates. However, Lab 2 exhibited a markedly inconsistent pattern: the net hydrogen consumption values (0.91 mmol and 0.99 mmol) were nearly half of the estimated values based on measured (1.56 mmol and 1.65 mmol) and theoretical (1.88 mmol and 1.96 mmol) sulfate concentrations. This discrepancy, together with other parameters such as 16 S rRNA gene copy numbers and pH evolution similar to other labs, suggests a significant underestimation of hydrogen consumption in Lab 2.

### Additional experiment on hydrogen consumption

Due to observed differences in microbial hydrogen consumption rates and abiotic hydrogen loss among the labs, two additional tests were conducted to measure hydrogen loss in distilled water under controlled conditions. Laboratories 1 and 3 optimized their experimental setups and repeated the abiotic loss test, achieving substantial improvements. The incorporated changes included (i) using thinner and smaller needles to pierce the stoppers. This led to smaller sampling losses and smaller holes, reducing leakage; (ii) using solid (not removable by hand) aluminum crimps to secure and tighten the stoppers, (iii) removing lubricant and powder from new stoppers by additional washing steps, vi) using a water bath to keep samples warm during pressure measurements. Following these optimizations, Lab 3 reported negligible hydrogen loss, while Lab 1 reduced its loss from an initial 26% (Online Resource 4, Figure S1) to approximately 5% (Online Resource 4, Figure S4). In addition, all four laboratories conducted a second simulated hydrogen consumption test to evaluate the precision of hydrogen consumption detection using a simulated hydrogen consumption protocol. The results showed strong inter-laboratory agreement, with a mean final hydrogen loss of 26.5% (SD = 1.65%) (Online Resource 4, Figure S3), confirming both the methodological improvements and the reliability of the hydrogen consumption data.

## Discussion

Previous studies on microbial hydrogen consumption in subsurface environments have primarily focused on isolated case studies (Hellerschmied et al. 2024; Keller et al. 2023; Mura et al. 2025) or laboratory-scale experiments using individual protocols (Mushabe 2025; Schwab et al. 2022), lacking standardization due to the relatively recent interest in hydrogen underground storage. While these efforts have provided valuable insights into microbial processes, they have not addressed the reproducibility and comparability of results across different research settings. To our knowledge, this is the first round-robin test conducted in the context of microbial hydrogen consumption in subsurface storage environments. By involving four independent laboratories and using a shared protocol with a defined reference strain, our study provides a benchmark for inter-laboratory consistency and highlights both the strengths and challenges of standardizing microbial activity assessments. We hope that this lays the groundwork for more harmonized methodologies in future hydrogen storage research projects.

In the performed round-robin test, we addressed several key aspects of monitoring microbial hydrogen consumption. Our primary objective was to develop and validate a standardized protocol for estimating microbial hydrogen consumption and net consumption rates, with the goal of ensuring broad applicability across diverse laboratory environments. This protocol was based on the collective experiences of individual labs and published data (Dopffel et al. 2023). The need for standardized methodologies is critical, as variations in microbial responses, analytical techniques, equipment, or environmental conditions can result in significant differences between the results. In addition to measurement of pressure and gas composition, we also monitored the changes in microbial abundances over time and analyzed the chemical composition of the original brine sample. Tracking key nutrients and gas concentration dynamics helps to identify the predominant microbial metabolism and the metabolic pathways responsible for hydrogen consumption in the tested systems.

### Hydrogen consumption by a sulfate-reducing standard strain

Our protocol proved suitable for estimating microbial hydrogen consumption in brine samples, and the overall trends were consistent. However, we still observed substantial variability in the hydrogen consumption between the labs. In the 9-day-long experiment, the estimated total consumption ranged from 19.8% (Lab 2) to 61% (Lab 4). Some of these differences can be attributed to the biological variability, a common phenomenon in microbiology (Delignette-Muller and Rosso 2000), as well as to differences in the methodological and analytical approaches. In exploring potential causes of observed variability, one possible explanation for the highest hydrogen consumption detected in Lab 4 is the highest original hydrogen content in the brine samples (~ 5 mmol) compared to other labs (~ 4.5 mmol). The hydrogen consumption curve follows an exponential trend, with the most rapid daily consumption occurring at the beginning of the experiment (Fig.1, Online Resource 3). Therefore, the higher initial hydrogen content likely accelerated early consumption until rising pH levels began to inhibit further activity (Dopffel et al. 2023). The high total hydrogen percentage consumption detected in Lab 4 is also consistent with other measured parameters. Notably, Lab 4 detected the most rapid increase in pH from the original brine pH 7 to pH over 9 in just two days of the experiment, and the most substantial sulfate reduction, with concentrations dropping from 1760 mg/L (or a theoretical 1852 mg/L) to 722 mg/L after nine days of incubation. When this sulfate loss is converted to theoretical hydrogen consumption using stoichiometric calculations and the ideal gas law, the results generally correspond well with observed hydrogen depletion, although the net hydrogen loss slightly exceeded theoretical expectations. However, Lab 4 also showed the greatest variability in estimated hydrogen consumption among replicates, particularly in control samples (sterile brine and sterile water). This suggests that limited analytical precision, likely due to manual sample loading during GC analysis, may have contributed to the observed differences. The rapid hydrogen consumption and the marked decrease in sulfate concentration provide strong evidence of active sulfate reduction, the primary metabolic pathway of the employed sulfate-reducing bacterium*Oleidesulfovibrio alaskensis* (Hauser et al. 2011).

The unexpectedly low hydrogen consumption observed in Lab 2 remains difficult to explain. Digital and digital droplet PCR (d/ddPCR) analyses indicated that microbial abundance was comparable across all brine samples, including those from Lab 2. Similarly, pH measurements in Lab 2’s hydrogen-incubated brine samples showed no significant deviation from those in other laboratories, although the theoretical effect of the brine carbonate/bicarbonate buffer system disruption during sample preparation must also be considered. Lastly, sulfate concentration decreased from 1700 mg/L to 910 mg/L, consistent with the other labs. This level of sulfate reduction should correspond to a hydrogen consumption of approximately 1.56 to 1.65 mmol. However, only a 0.91 to 0.99 mmol hydrogen decrease was recorded. Given the consistency in sulfate reduction, pH increase, and 16 S rRNA gene copy numbers across all labs, the anomalously low hydrogen consumption observed in Lab 2 might be attributed to analytical inaccuracies in hydrogen or pressure measurements during the experiment.

To investigate this possibility, we conducted a control experiment simulating biotic hydrogen loss in sterile water by manually extracting a consistent volume of gas before each measurement. The results were consistent across all labs, with only minor variability, confirming the reliability of hydrogen monitoring procedures across the laboratories.

The variability in hydrogen consumption observed in our single-strain, round-robin test reflects the natural differences that can arise when protocols are applied across laboratories with different analytical setups, sample handling, and measurement precision (Youden 1969). For modeling of microbial hydrogen consumption, which has been attempted before (Gao et al. 2024; Strobel 2023), such variability can be effectively addressed by using mean hydrogen consumption rates or incorporating observed minimum and maximum values. To ensure a more comprehensive assessment of microbial activity, we recommend complementing hydrogen measurements with additional indicators such as sulfate reduction, pH shifts, and the production of typical metabolites, which indicate hydrogen-consumption activity like H₂S, acetate, or methane (Gregory et al. 2019).

### Abiotic hydrogen loss and protocol optimization

One of the key parameters for accurately estimating microbial hydrogen consumption is quantifying abiotic hydrogen loss, particularly due to leakage of hydrogen through the butyl rubber stoppers (Nauer et al. 2021), which can lead to overestimating the microbial consumption. This loss can be quantified using sterile water incubated with hydrogen at the same temperature as the brine samples, and the values can be used to adjust the observed hydrogen consumption rates. We have observed marked differences in abiotic loss between the labs. No or negligible loss was observed in Lab 4 and Lab 2, respectively. This parameter thus did not contribute to the marked differences in hydrogen consumption between Labs 2 and 4. On the other hand, considerable abiotic hydrogen loss was detected in Lab 3 and Lab 1, 8.8% in 9 days and 26% in 9 days, respectively. Significant improvements were achieved by using thinner needles, enhancing stopper tightness by removing all surface preservatives, and maintaining stable temperatures during pressure measurements, resulting in a maximum abiotic loss of only 5%.

Overall, our test revealed the crucial role of abiotic controls (at all studied temperatures) in measuring the hydrogen loss caused by sampling and leaking through the stoppers. Our data clearly demonstrates that neglecting such controls can result in a severe overestimation of microbial hydrogen consumption.

### Test brine chemical composition

The chemical composition of the original brine samples was analyzed to estimate the concentration of various nutrients that support microbial metabolism. In the round-robin, we focused only on the compounds relevant to our simplified artificial brine system, but a much broader analytical spectrum is advisable (Table 1) in the case of real brine samples to capture the whole possible diversity of hydrogen-consuming microbes. The comparative analysis demonstrated good consistency in identified test brine composition before the incubation reported by individual laboratories, except for aqueous sulfide detection, where the measured values were significantly lower than expected across all laboratories. Although all the aqueous sulfide results except for Lab 3 were obtained in the ISO 17,025-accredited laboratories, the observed variability was significant. Possible explanations for the discrepancy are manifold due to the inherent difficulty of measuring hydrogen sulfide and aqueous sulfide in natural systems (Lawrence et al. 2000; Shen et al. 2012). Sulfide is a highly reactive, pH-sensitive analyte, and accurate quantification is challenging. Possible explanations for the observed differences include sulfide precipitation and scaling during sample handling, chemical reactivity of sulfide leading to concentration changes, inappropriate sample storage, resulting in degassing, and subsequent sulfide loss. The brine chemical composition was checked for the potential of sulfide scaling using commercial ScaleSim software (scalesim.com), but it could only contribute to a maximum loss of 0.5 mg/L of aqueous sulfide. Immediate analysis upon sample arrival and improved handling protocols are likely necessary to enhance the reliability of sulfide measurements in the future. Further testing is therefore recommended to identify best practices and minimize analytical variability.

### Interpreting pH and chemical shifts during brine incubations

In addition to the test brine analysis before the incubation, we also focused on changes in brine chemical composition during incubations and H_2_S production. Our data confirmed that the pH change during the experiment is a good indicator of ongoing microbial hydrogen consumption. In the case of organotrophic microbial activity, acetate or CO_2_ are typically produced by the oxidation of the organic compounds, which might lead to acidification and pH decrease depending on the solution’s buffering capacity (Wang et al. 2018). On the other hand, the consumption of H_2_ leads to a pH increase due to intense proton consumption (Dopffel et al. 2023). In agreement with this, a rapid and massive increase in pH was observed in all living brine samples incubated with hydrogen, while only minute pH changes were observed in sterile brine samples or brine samples incubated with nitrogen. However, the pH increase observed in Lab 2 without a corresponding decrease in hydrogen concentration demonstrates that interpreting pH shifts needs to be done cautiously, as microbial activity alone probably has not accounted for the observed change. Like many environmental samples, the artificial brine used in the experiments was buffered with a bicarbonate/CO₂ system (Boyd 2000). During preparation, the headspace was flushed with 100% H₂. If this gas exchange is performed too abruptly, it can lead to CO₂ stripping from the headspace, triggering bicarbonate degassing and a subsequent rise in pH. This abiotic mechanism is supported by PHREEQC calculations performed to obtain theoretical artificial brine composition, which showed that CO₂ outgassing during sampling could elevate the pH from 7.50 (as measured in the reactor, at 37 °C) to as high as 9.0 under equilibrium with atmospheric conditions, at 25 °C. Therefore, the pH increase observed in Lab 2, can theoretically also be attributed to this abiotic effect. These findings highlight the importance of considering carbonate system dynamics when interpreting pH changes in hydrogen incubation experiments. To improve data reliability, we recommend (1) standardizing the H₂/N₂ flow rates during headspace flushing, (2) monitoring pH immediately after gas flushing, and (3) including proper controls (e.g., brine incubated with N₂) to distinguish between biological and chemical contributions to pH variation. An alternative option is to consider the use of an organic buffer system to stabilize pH. However, adding such a buffer would significantly alter the sample’s chemical composition and is therefore not advisable when working with authentic environmental brines (Ferreira et al. 2015).

In addition to pH monitoring, we also tracked changes in acetate, sulfate, and H_2_S concentrations in the living brine samples during the incubation. Acetate represents a primary organic carbon source and sometimes also an electron donor for SRB metabolism (Muyzer and Stams 2008), while sulfate is the key electron acceptor in our tested experimental system. Ongoing microbial sulfate reduction was detected in brine samples incubated with hydrogen, and the sulfate decrease corresponded reasonably well to the observed hydrogen consumption, except for Lab 2. On the other hand, measurements of gaseous H_2_S concentrations revealed only negligible levels of this compound, despite ongoing sulfate reduction. This is likely due to the rapid pH increase observed in the brine, resulting in dissolution of the hydrogen sulfide in the brine (Millero 1986). Unfortunately, aqueous HS⁻ was not analyzed to confirm this hypothesis due to the limited sample volume. Further improvements in this compound detection from very low sample volumes are thus needed.

The samples with nitrogen did not show consumption of acetate or sulfate, or production of H_2_S during the experiment. This indicates that sulfate reduction with acetate as the electron donor is not performed by the studied *Oleidesulfovibrio alaskensis* strain, which is consistent with previously published data (Feio et al. 2004; Waite et al. 2020). However, we observed a minute decrease in acetate concentration in brine samples incubated with hydrogen in Lab 2, Lab 3, and Lab 4 (Lab 1 data not considered due to analytical problems). As the microbial abundance was several times higher in brine samples incubated with H_2_ compared to N_2_, it is clear that *Oleidesulfovibrio alaskensis* uses acetate solely as a carbon source.

**Table 7:** Recommendations for standardizing hydrogen consumption experiments. IST = in-situ temperature

Key points	Details
Sample Collection	Obtain fresh brine samples specifically for microbiological testing with specific care to keep the brine anoxic.
Temperature Conditions	Conduct experiments at the current in situ temperature (IST) of the natural brine storage site to ensure physiological relevance and accurate microbial activity assessment.
Experimental Controls (at IST)	- Natural brine + H₂ at IST - Sterilized brine + H₂ at IST - Sterile distilled water + H₂ at IST
Standardized Controls (for comparison)	- Natural brine + H₂ at 30 °C - Sterile distilled water + H₂ at 30 °C (to estimate abiotic loss)
Complementary Data for Interpretation	Monitor the following parameters to properly interpret microbial hydrogen consumption: - Sulfate concentrations - Gaseous products (e.g. CH₄, H₂S) - Liquid products (e.g. acetate, formate, sulfide) - pH changes - Cell number increase
Abiotic Hydrogen Leakage Consideration	Each gas or liquid sample must be accompanied by pressure measurements at the correct temperature to calculate sampling loss and avoid overestimating microbial activity.
Documentation	Clearly document: - Incubation temperatures - Liquid and headspace volumes used - Control conditions used in each experiment to support reproducibility and comparability.

### Conclusions and impact

Standardized protocols are crucial for producing reliable, high-quality data, which can be compared between labs and projects. Our developed protocols enable direct assessment of microbial hydrogen consumption in original brine samples, combining gas, chemical, and genetic analyses. The key elements for effective standardization are summarized in Table 7, providing a comprehensive view of microbial activity and associated metabolic processes within the storage site.

The round-robin validation across four laboratories demonstrated the protocol’s robustness and reproducibility in detecting microbial H₂ consumption trends. Still, despite the overall consistent trend, quantitative variability in H₂ consumption across labs (19.8–61%) highlights the influence of analytical methods, sample handling, and biological variability. Abiotic hydrogen loss, particularly due to leakage from experimental bottles, remains a significant challenge and can impact microbial consumption estimates. These findings highlight the critical importance of controlling experimental conditions and carefully evaluating each step to minimize abiotic loss. Such considerations should be integrated into future projects and modeling efforts.

It is important to note that the protocol may not fully capture slow-growing or low-abundance microbial populations, which could be relevant in long-term storage scenarios. In such cases, methods and incubation times need to be adapted individually. Additionally, although the use of artificial brine in inter-lab comparisons facilitates standardization, it may not fully replicate the complexity of natural field conditions. Further studies focusing on comparisons using real brine samples are therefore necessary.

Overall, we recommend complementing hydrogen measurements with monitoring of additional microbial activity indicators, such as pH changes, sulfate reduction, and production of H₂S, acetate, or methane. These these parameters can provide valuable contextual information. In our round-robin study, these indicators correlated well with hydrogen consumption, further supporting the protocol’s robustness.

A notable strength of the developed protocols is their flexible design, which allows inclusion of additional microcosms under varying conditions. This flexibility makes the protocol a valuable tool for investigating hydrogen consumption kinetics and rates across different experimental setups. While further validation is needed across a broader environmental context, our proposed protocols have the potential to enhance data comparability between studies and contribute to a more consistent understanding of microbial processes in underground hydrogen storage systems.

## Supplementary Information

Below is the link to the electronic supplementary material.

Supplementary Material 1

Supplementary Material 2

Supplementary Material 3

Supplementary Material 4

## Data Availability

The hydrogen consumption datasets generated and analyzed during this study are available as Online Resource 3. Other data are presented in graphical form within the manuscript as online supplementary material, and the corresponding source data are available from the corresponding author upon request.
