# Supplementary material for: Towards standardized microbial hydrogen consumption testing in the subsurface: harmonized field sampling and enrichment approaches
Source: World J Microbiol Biotechnol. 2025 Sep 26;41(10):342. doi: 10.1007/s11274-025-04542-0 (PMC12464130; doi:10.1007/s11274-025-04542-0)
Supplement: Supplementary file 1 — Supplementary Material 1 [file 11274_2025_4542_MOESM1_ESM.docx]

**Towards standardized microbial hydrogen consumption testing in the subsurface: Harmonized field sampling and enrichment approaches**

Kateřina Černá^1*^, Kristýna Fadrhonc^1^, Jakub Říha^1^, Petra Bombach^2^, Sylvain Stephant^3^, Caroline Michel^3^, Laura Fablet^3^, Joachim Tremosa^4^, Kyle Mayers^5^, Biwen Annie An-Stepec^5^, Nicole Dopffel^5^

1 Technical University of Liberec, Institute for Nanomaterials, Advanced Technologies and Innovation, Bendlova 7, 46117 Liberec, Czechia

2 Isodetect GmbH, Deutscher Platz 5b, 04103 Leipzig, Germany

3 BRGM, 3 Avenue Claude Guillemin, 45060 Orléans Cedex 2, France

4 Geostock, 2 Rue des Martinets, 92500 Rueil-Malmaison, France

5 Norwegian Research Centre AS – NORCE, Nygårdsgaten 112, 5008 Bergen, Norway

*Corresponding author: [katerina.cerna1@tul.cz](mailto:katerina.cerna1@tul.cz), ORCID ID: 0000-0003-3351-6372


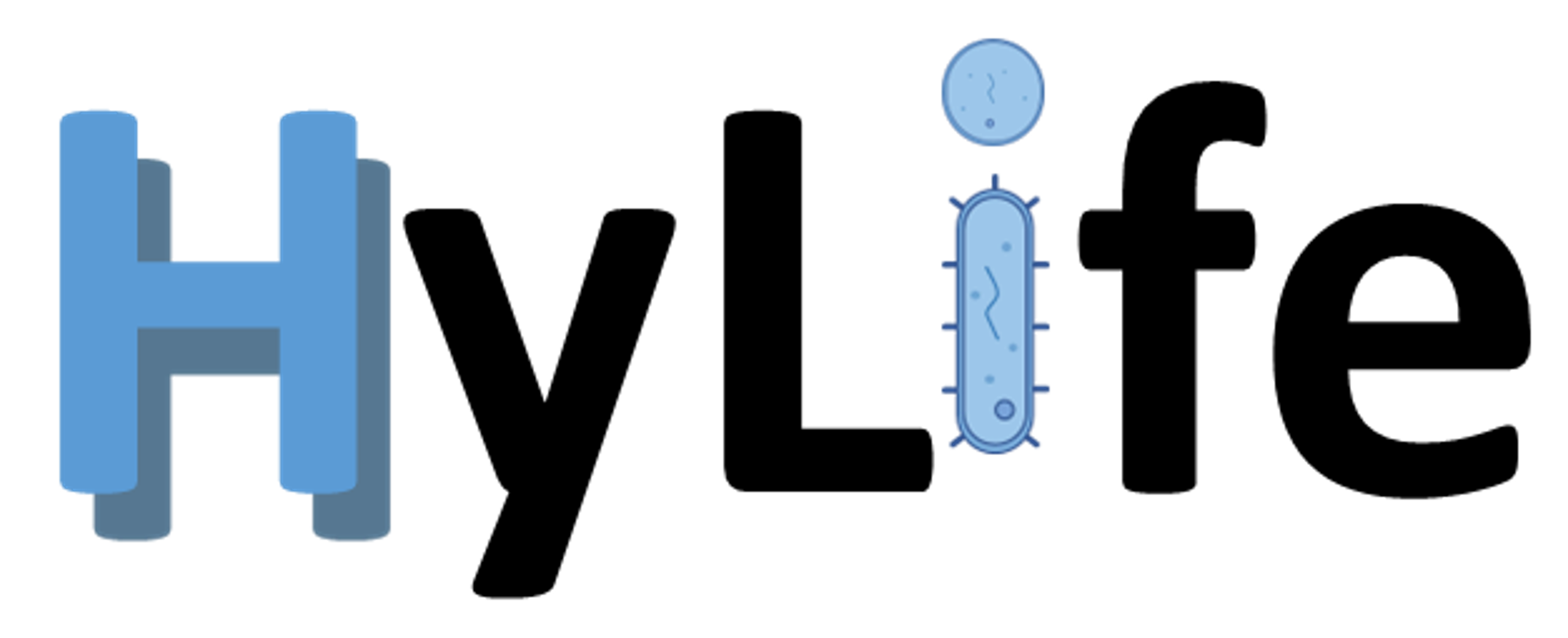

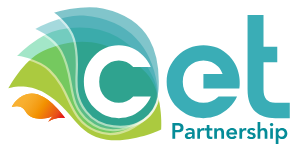


2024

v.2

Standard operational procedures for sampling of water from gas storage reservoirs

Water sampling using N2 gas

**CETP Project HyLife SOP**

Table of contents

[1. Material supply description 1](#_Toc152753475)

[2. Some explanation 2](#_Toc152753476)

[3. Water sampling description 2](#_Toc152753477)

[a) Step 1: Preparing the sample bottle filled with nitrogen 3](#_Toc152753478)

[b) Step 2: Water sample 3](#_Toc152753479)

[c) Step 3: Finish 5](#_Toc152753480)

[4. Sample shipment 5](#_Toc152753481)

## Material supply description

*This material will be sent from the lab to the field:*

- 2 x pressure-resistant (picture A – Figure 1), anaerobic (N_2_ or argon flushed) 1L each, sterile (autoclaved) glass bottles closed with thick butyl rubber stopper (picture B – Figure 1) and a red crew cap (picture A – Figure 1).
- Tubing with sterile filter (0.2 µm) for flushing the sample with nitrogen - sterile packed in aluminum foil or autoclavable / sterile plastic bags (picture D – Figure 1).

!!! The diameter of pressure reducer outlet needs to be discussed beforehand!!!

- Tubes for water sampling (picture C – Figure 1) including the connectors to attach the tubing to the tap, sterile packed in aluminum foil or autoclavable plastic bags/ sterile plastic bags.
- Sterile stock of butyl stoppers (picture B – Figure 1), single packed (to be used in case of stopper contamination during sampling)
- Several garbage bags (picture F – Figure 1)
- Styrofoam boxes for sampling bottles transport (picture A – Figure 1).
- Laboratory gloves
- Disinfectant wipes or similar for wiping and gloves (picture E – Figure 1).

*This material needs to be provided by the field operator:*

- N_2_ or Ar gas pressure bottle. Better N2 quality of advantage (5.0, 99.99% N_2_ or Ar).


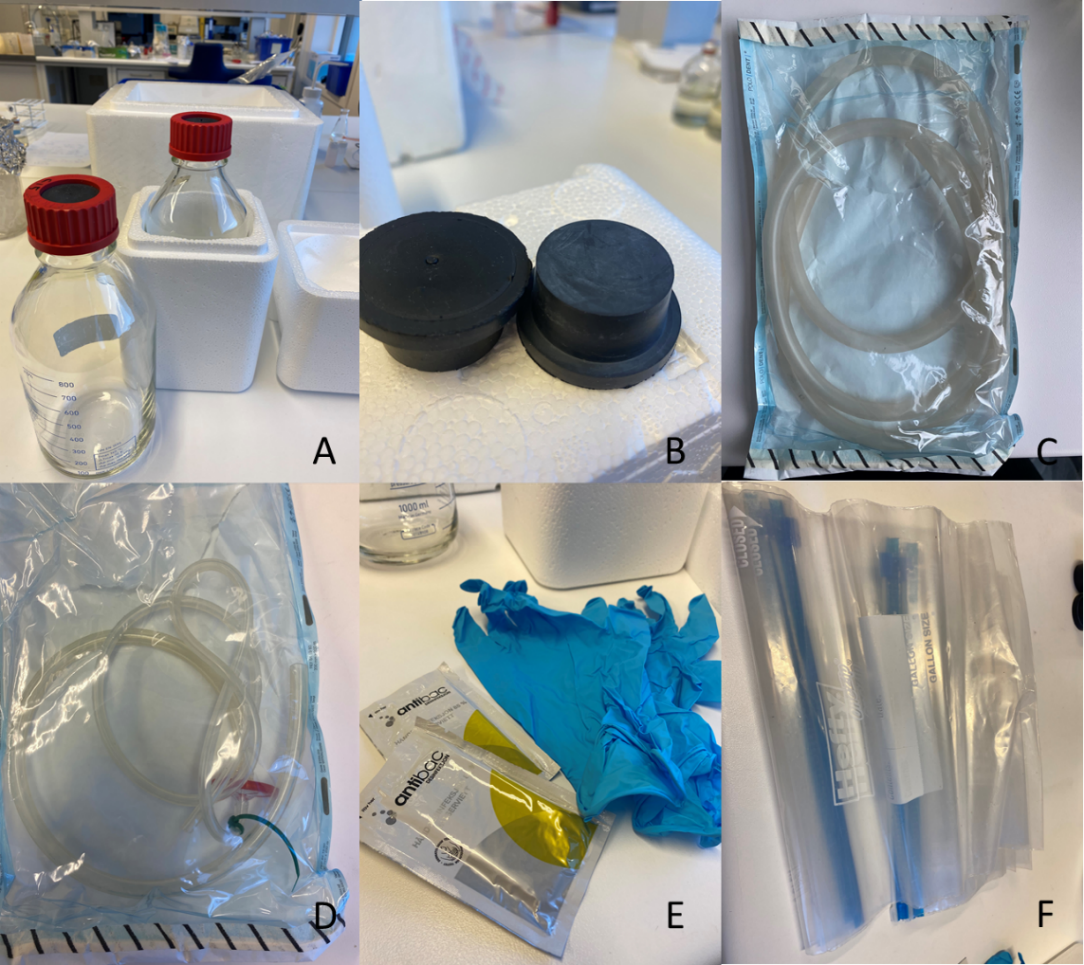


Figure 1: Supplies for water sampling: (A) Sterile pressure-resistant sample bottle packed in Styrofoam boxes, (B) rubber stoppers, (C) sterile tubing for water sampling (no filter attached), (D) sterile tubing with sterile filter for nitrogen flushing, (E) disinfectant wipes and gloves, (F) garbage bags.

## Some explanation

**Tube with sterile filter for N_2_ fumigation:**

The tubes are used to flush the sample bottle with N_2_. It needs to be clarified beforehand which connection the N_2_ bottle has. The tubes are provided sterile by the lab.

The tubes can be used for the entire sampling. Wipe the valve between different sampling points with ethanol/wipes

**Tubes for sampling:**

The tubes are used to sample the water from the tap. Approximately 2-meter tubing including necessary connectors to fit the outlet valve at the extraction point are provided sterile by us.

Use tubes only once

## Water sampling description

Best to be done with 2 people! One person takes care of the permanent N_2_ flushing during sampling. The other performs the actual sampling.

!!! Wear gloves and sterilize them with the wipes! Don't open anything beforehand, but always right before use!!!

**The whole sampling protocol is summarized in a short video:**

<https://youtu.be/e0utSDKVImk>

**Important note: We need two 1l bottles filled with water at the end of sampling procedure, so you need to performed following procedure twice independently.**

1. Step 1: Preparing the sample bottle filled with nitrogen
2. Prepare all the sampling equipment (Figure 1) and keep it ready to use.
3. Connect the tubing with sterile filter to the N_2_ gas bottle.

!!! Do not touch the free end of the tubing!!!

1. Open the N_2_ gas bottle and rinse the tubes with N_2_ for a 2 minutes.

!!! Make sure N_2_ really flows (e.g. skin test - point the tube tip on the skin of your forearm without touching your skin and you should feel the flow)!!!

1. Meanwhile, prepare the sample bottle with the **red screw cap,** unscrew cap and remove the butyl stopper carefully from the bottle, hold it without touching the bottom part or deposit it on sterile aluminium foil.

!!! In case of putting the butyl stopper on the aluminium foil always face up!!!

1. Slide the free end of the tubing inside the sample bottle while still flushing it with the nitrogen.

!!! Do not touch the free end of the tubing, you can only touch the tubing between the N_2_ bottle and the filter!!!

1. Put the stopper back on the bottle loosely (Figure 2). Flush the bottle for 2 minutes and continue with N_2_ flushing until the water sampling (next section)_._

!!! Do not touch the bottom part of the stopper. Take new sterile stopper from the stock if the bottom part is contaminated!!!


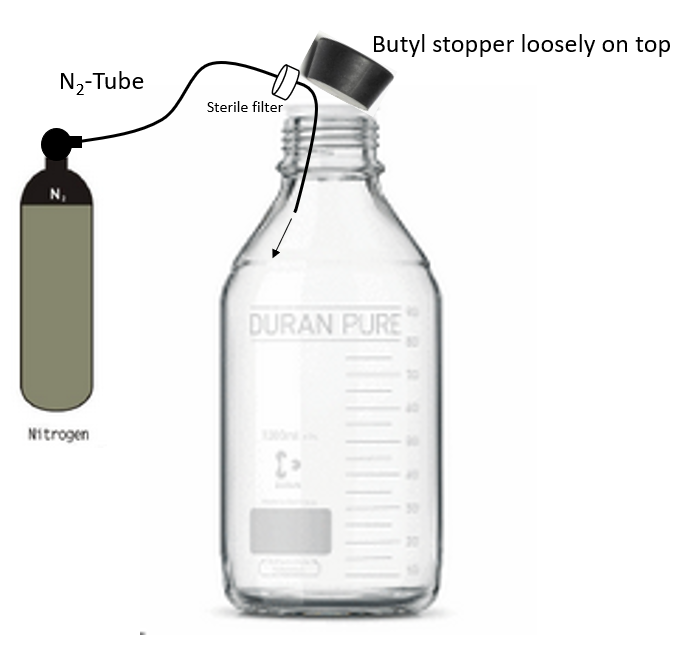


Figure 2: Flushing of the sample bottle with nitrogen.

### Step 2: Water sample (**2 x 1 L bottle**)

1. Open the tap and let the water run first freely for some time 5-10 minutes (discard directly or first in bucket and then discard). The water standing in the pipe needs to be removed first. The water flushing should represent three times the volume water standing.
2. Close the tap and wipe the tap with disinfectant wipes.
3. Connect the sampling tube with the outlet tap valve.

!!! Do not touch the free end of the tube!!!

1. Open the tap and let some water run through the sampling tubing and discard it.
2. Remove the butyl stopper carefully from the bottle while still flushing with N_2_, hold it without touching the bottom part or deposit it on sterile aluminium foil.

!!! In case of putting the butyl stopper on the aluminium foil always face up!!!

1. Slip the free end of the sampling tube inside the sampling bottle without touching the free end of the tube. Let the water run to the bottom the of the bottle along the glass wall to avoid air ingress (Figure 3).
2. Fill the sample with 1L of water under constant N_2_ flush (fill the bottle to 80% = shoulder of bottle - the water should reach the level visualized in Figure 4). The gas should bubble through the water all the time.


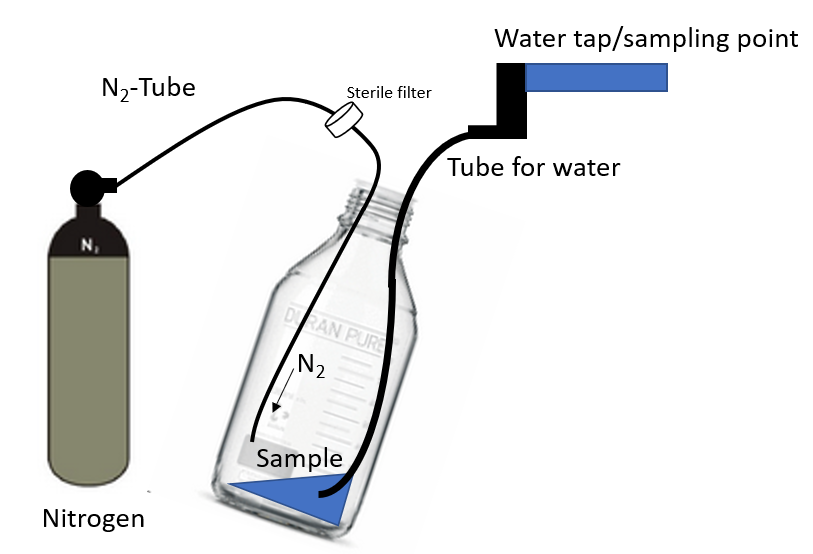


Figure 3: Water sampling.

1. Stop water flow, take the sampling tube out while still flushing the bottle with N_2_ **(the gas should bubble through the water all the time)**.
2. Put butyl stopper slightly on bottle while still flushing the bottle with N_2,_ Figure 4.

!!! Take new sterile stopper from the stock if you have doubts about the sterility of the stopper!!!

1. Continue flushing the liquid for 2-3 minutes with N_2_ so all air is removed from gas headspace above the water.


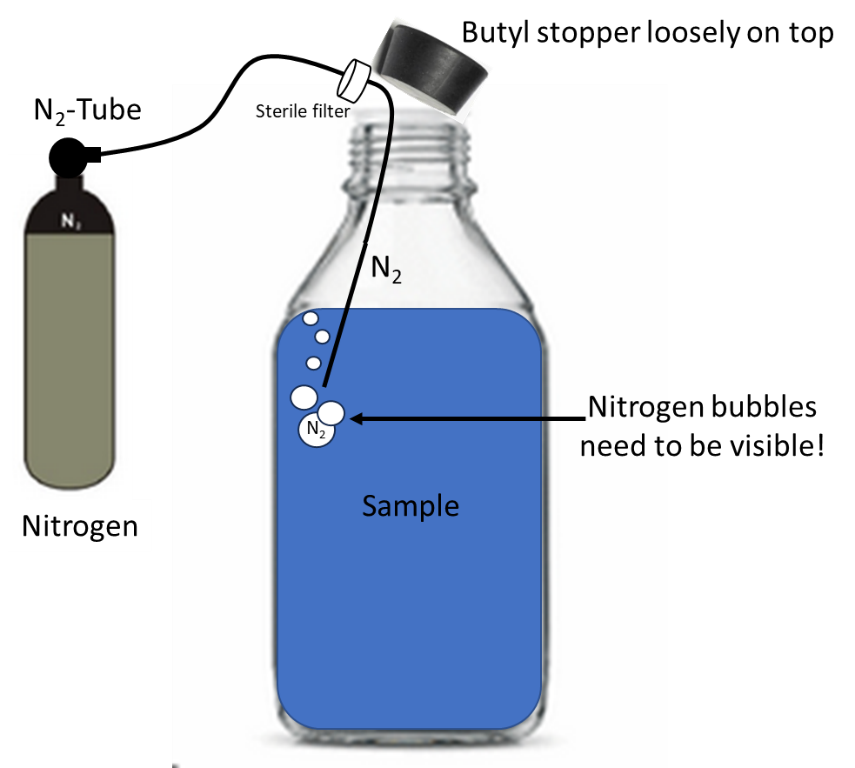


Figure 4: Flushing the bottle with water sample with nitrogen.

1. Remove the N_2_ tube from the bottle and **press the butyl stopper in immediately** and firmly.
2. Screw on the bottle red cap and make sure everything is tightly closed.
3. Take the photo of freshly samples water and if possible, imediatelly send the photo to the laboratory, which will process the sample, to check the quality.
4. **Repeat procedures described in Step 1 and Step 2 so you have 2 x 1 L bottles filled with brine at the end of sampling.**

### Step 3: Finish

1. Close N_2_ bottle.
2. Wipe the outside of the bottles dry if they have become wet.
3. Label the bottle (sample name, location, company, date)
4. Pack labeled bottles in prepared styrofoam boxes.
5. Pack all used tubes in provided garbage bags and put it back to the transport box (some parts can be resued after sterilization).

**
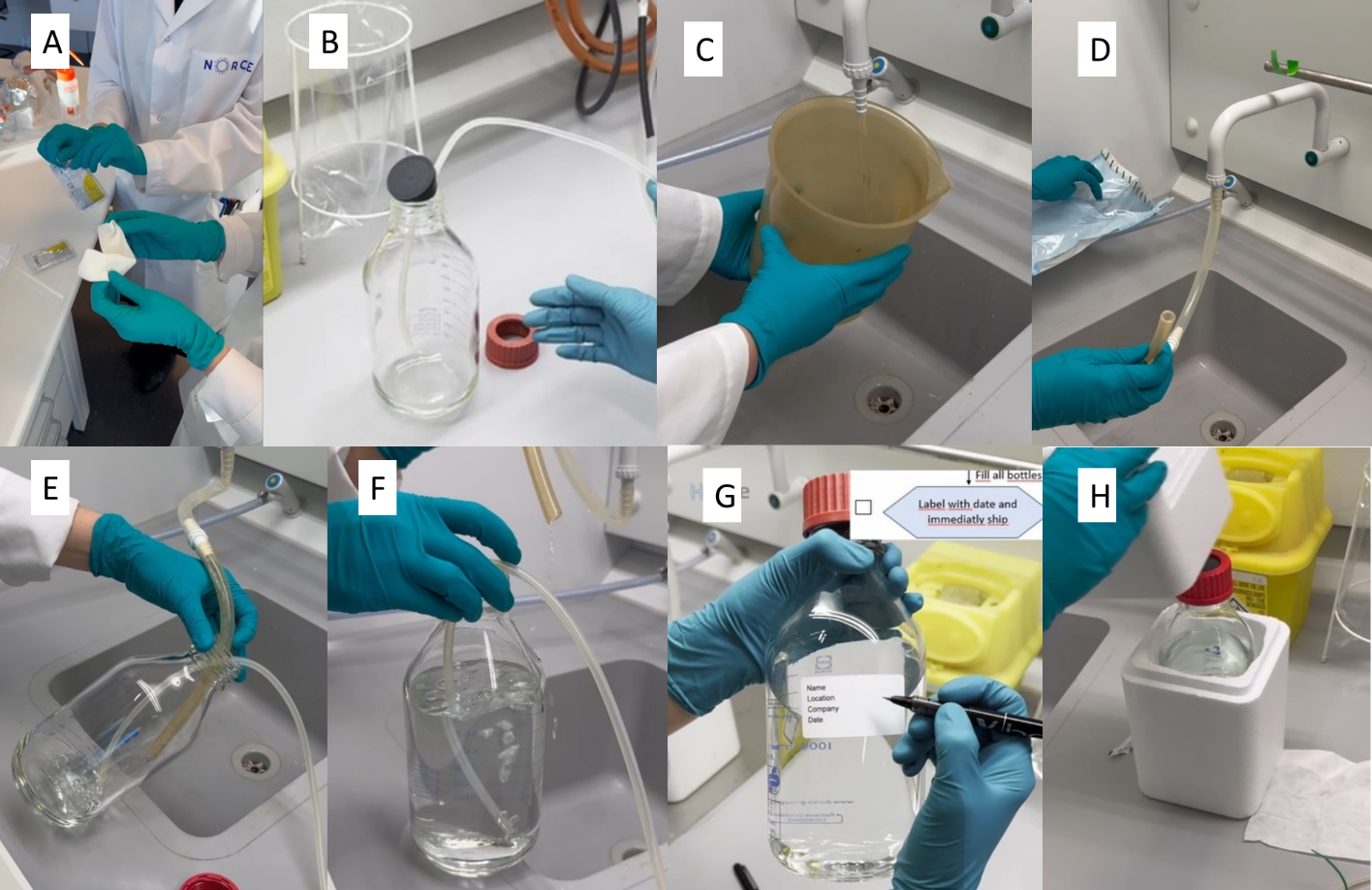
**

Figure 5: Sampling overview: (A) Wear gloves and sterilize them before you start. (B) Flush the provided glass bottle with the nitrogen. (C) Flush the pipes properly before sampling. (D) Connect the tubing to the tap. (E) Sample the water while continuously flushing the bottle with nitrogen. (F) Fill the bottle to 2/3 volume and continue flushing with nitrogen. (G) Close the bottle and write the label. (H) Pack the bottle into a styrofoam box.

## Sample shipment

1. After collection, sample bottles with samples should be kept in the Styrofoam boxes in the dark at room temperature. Avoid unnecessary delay in the sample shipment.
2. Ship the samples to the lab safely packed in the Styrofoam boxes.
