# Supplementary material for: Towards standardized microbial hydrogen consumption testing in the subsurface: harmonized field sampling and enrichment approaches
Source: World J Microbiol Biotechnol. 2025 Sep 26;41(10):342. doi: 10.1007/s11274-025-04542-0 (PMC12464130; doi:10.1007/s11274-025-04542-0)
Supplement: Supplementary file 3 — Supplementary Material 3 [file 11274_2025_4542_MOESM3_ESM.docx]

**Towards standardized microbial hydrogen consumption testing in the subsurface: Harmonized field sampling and enrichment approaches**

Kateřina Černá^1*^, Kristýna Fadrhonc^1^, Jakub Říha^1^, Petra Bombach^2^, Sylvain Stephant^3^, Caroline Michel^3^, Laura Fablet^3^, Joachim Tremosa^4^, Kyle Mayers^5^, Biwen Annie An-Stepec^5^, Nicole Dopffel^5^

1 Technical University of Liberec, Institute for Nanomaterials, Advanced Technologies and Innovation, Bendlova 7, 46117 Liberec, Czechia

2 Isodetect GmbH, Deutscher Platz 5b, 04103 Leipzig, Germany

3 BRGM, 3 Avenue Claude Guillemin, 45060 Orléans Cedex 2, France

4 Geostock, 2 Rue des Martinets, 92500 Rueil-Malmaison, France

5 Norwegian Research Centre AS – NORCE, Nygårdsgaten 112, 5008 Bergen, Norway

*Corresponding author: [katerina.cerna1@tul.cz](mailto:katerina.cerna1@tul.cz), ORCID ID: 0000-0003-3351-6372

The tables below summarize the analytes and analytical approaches applied by individual labs during round-robin tests.

| **Lab 1** | **Methods** | **Volume taken** | **unit** | **Sample preservation** |
| --- | --- | --- | --- | --- |
| **ph** | LAQUA twin pH compact (Horiba​) | 0.5 mL |  | directly processed |
| **Salinity** | LAQUA twin conductivity compact (Horiba​) |  | % | directly processed |
| **Eh** | LAQUA twin Eh compact (Horiba​) |  | mV | directly processed |
| **Pressure** | Keller manometer (connect to the GC) | 500 µL | mbar | directly processed |
| **Gas composition** | GC |  | % | directly processed |
| **Inorganic anions** | F, Cl, NO3, SO4, Br: IC  PO4: Spectrophotometry | 100 mL | mg/L | sterile filtered, stored in the fridge |
| **Dissolved HS^-^** | In-house photometric method | 125 mL | mg/L | fixation with zinc acetate and HO-, stored in the fridge |
| **Cations** | Ca, K, Mg, Na: ICP-AES Mn: ICP-MS NH4, NO2: Spectrophotometry | 50 mL | mg/L | sterile filtered, acidified with HNO3, stored in the fridge |
| **Organics compounds** | Performed by Lab 3 (HPLC) | 1 mL | mg/L | frozen at -20°C |
| **Total dissolved organic carbon (DOC)** | NF EN 1484 | 125 mL | mg/L | sterile filtered and acidified with H2SO4, stored in the fridge |
| **Total inorganic carbon (TIC)** | Sodium persulfate oxydation | 125 mL | mg/L | stored in fridge |
| **16S copy number** | dPCR at Lab4 | 1 mL | cells/L | pellets frozen until extraction with Blood and Tissue Kit |

| **Lab 2** | **Methods** | **Volume taken** | **unit** | **Sample preservation** |
| --- | --- | --- | --- | --- |
| **pH** | Open membrane type pH meter - LAQUA twin pH compact (Horiba​)/ WTW (electrode system) | 0.3 mL |  | directly processed |
| **Salinity** | Pocket Salt meter ATAGO​/WTW (electrode system) | 0.5 mL | % | directly processed |
| **Eh** | n.a. | n.a. | n.a. | n.a. |
| **Pressure** | Pocket Instrument Greisinger/Small manual manometers |  | mbar | directly processed |
| **Gas composition** | GC-ECD/WLD | 30 µl | % | directly processed |
| **Inorganic anions** | external lab; Ion Chromatography; DIN EN ISO 10304-01 (D 20) | 100 mL | mg/L | fast handover to the laboratory |
| **Dissolved HS^-^** | in house: photometric method,  external lab: DIN 38405-27 | 0.2 mL 100 mL for inorganic anions | mg/L | fixation with zinc acetate |
| **Cations** | external lab: ICP; DIN EN ISO 11885 (E 22) | 50 mL | mg/L | fast handover to the laboratory |
| **Organics compounds** | Performed by Lab 3 (HPLC) | 1 mL | mg/L | frozen at -20°C |
| **Total dissolved organic carbon (DOC)** | external lab: DIN EN 1484 (H 3) | 50 mL | mg/L | fast handover to the laboratory |
| **Total inorganic carbon (TIC)** | external lab: DIN EN 1484 (H 3) | 100 mL | mg/L | fast handover to the laboratory |
| **16S copy number** | dPCR at Lab4 | 1 mL | cells/L | pellets frozen until extraction with Blood and Tissue Kit |

| **Lab 3** | **Methods** | **Volume taken** | **Unit** | **Sample preservation** |
| --- | --- | --- | --- | --- |
| **pH** | Open membrane type pH meter - LAQUA twin pH compact (Horiba​)/ WTW (electrode system) | 0.3 mL |  | directly processed |
| **Salinity** | Pocket Salt meter ATAGO​/WTW (electrode system) | 0.5 mL | % | directly processed |
| **Eh** | WTW (electrode system) | 1 mL | mV | directly processed under anoxic conditions |
| **Pressure** | Pocket Instrument Greisinger/Small manual manometers |  | mbar | directly processed |
| **Gas** **composition** | microGC Agilent 490 | 500 uL | % | directly processed |
| **Inorganic anions** | IC/ICP external ISO 11885 | 10 mL | mg/L | sterile filtered |
| **Dissolved HS^-^** | photometric Cline assay | 200 µl | nmol | stabilized in zinc acetate, frozen |
| **Cations** | IC/ICP external ISO 11885 | 10 mL | mg/L | fixed with HNO3 to pH <2, |
| **Organics compounds** | HPLC | 1 mL |  | frozen at -20°C |
| **Total dissolved organic carbon (DOC)** | Elementar vario TOC cube | 10 mL | mg/L | sterile filtered, stored in the fridge |
| **Total inorganic carbon (TIC)** | Elementar vario TOC cube | 10 mL | mg/L | sterile filtered, stored in the fridge |
| **16S copy number** | ddPCR | 1 mL | cells/L | pellets frozen until extraction with Blood and Tissue Kit |

| **Lab 4** | **Methods** | **Volume taken** | **unit** | **Sample preservation** |
| --- | --- | --- | --- | --- |
| **pH** | Open membrane type pH meter - LAQUA twin pH compact (Horiba​)/ WTW (electrode system) | 0.7 mL |  | directly processed |
| **Salinity** | Pocket Salt meter ATAGO​/WTW (electrode system) |  | % | directly processed |
| **Eh** | WTW (electrode system) | 10 mL | mV | directly processed under anoxic conditions |
| **Pressure** | Pocket Instrument Greisinger/Small manual manometers |  | mbar | directly processed |
| **Gas composition** | gas chromatography (Clarius 580-FID/TCD for CH4,CO2 and H2S, Thermo Trace 1310, Thermo TCD for H2) | 200 µl for H2 500 µl for CO2 and CH4 | % | directly processed |
| **Inorganic anions** | liquid chromatography (Dionex ICS 90, ThermoFisher Scientific), PO4: Spectrophotometry (ČSN EN ISO 6878, SM 4500-P), external | 10 mL | mg/L | sterile filtered |
| **Dissolved HS^-^** | Spectrophotometry (ČSN 83 0520-16:1978, ČSN 83 0530-31:1980, SM 4500-S2- D), external | 60 mL | mg/L | fixed with zinc acetate |
| **Cations** | metals ICP OES, PE 2100DV/ NH4+ spectrophotometry (Hach cuvette system) | 15 mL | mg/L | fixed with HNO3 to pH <2, stored in the fridge |
| **Organics compounds** | Capillary electrophoresis with UV detection | 1 mL | mg/L | frozen at -20°C |
| **Total dissolved organic carbon (DOC)** | IR detection (ČSN EN ISO 20236, SM 5310), external | 60 mL | mg/L | fixed with HCl, stored in the fridge |
| **Total inorganic carbon (TIC)** | IR detection (ČSN EN ISO 20236, SM 5310), external | 15 mL | mg/L | stored in fridge |
| **16S copy number** | dPCR (QIAcuity Digital PCR System) | 1 mL | cells/L | pellets frozen until extraction with Blood and Tissue Kit |
