# Supplementary material for: Towards standardized microbial hydrogen consumption testing in the subsurface: harmonized field sampling and enrichment approaches
Source: World J Microbiol Biotechnol. 2025 Sep 26;41(10):342. doi: 10.1007/s11274-025-04542-0 (PMC12464130; doi:10.1007/s11274-025-04542-0)
Supplement: Supplementary file 4 — Supplementary Material 4 [file 11274_2025_4542_MOESM4_ESM.docx]

**Towards standardized microbial hydrogen consumption testing in the subsurface: Harmonized field sampling and enrichment approaches**

Kateřina Černá^1*^, Kristýna Fadrhonc^1^, Jakub Říha^1^, Petra Bombach^2^, Sylvain Stephant^3^, Caroline Michel^3^, Laura Fablet^3^, Joachim Tremosa^4^, Kyle Mayers^5^, Biwen Annie An-Stepec^5^, Nicole Dopffel^5^

1 Technical University of Liberec, Institute for Nanomaterials, Advanced Technologies and Innovation, Bendlova 7, 46117 Liberec, Czechia

2 Isodetect GmbH, Deutscher Platz 5b, 04103 Leipzig, Germany

3 BRGM, 3 Avenue Claude Guillemin, 45060 Orléans Cedex 2, France

4 Geostock, 2 Rue des Martinets, 92500 Rueil-Malmaison, France

5 Norwegian Research Centre AS – NORCE, Nygårdsgaten 112, 5008 Bergen, Norway

*Corresponding author: Kateřina Černá, [katerina.cerna1@tul.cz](mailto:katerina.cerna1@tul.cz), ORCID ID: 0000-0003-3351-6372


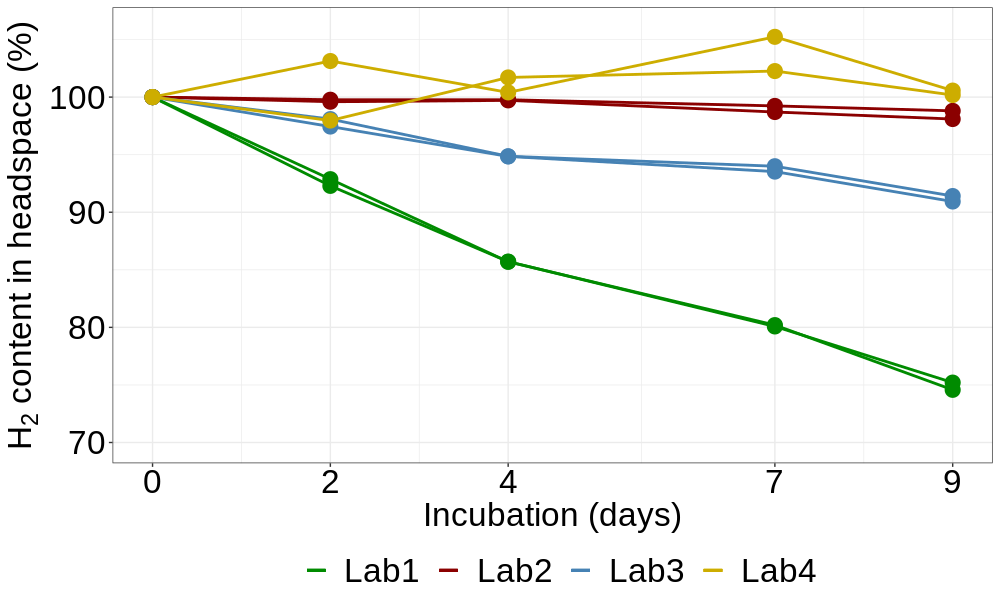


Supplementary Figure S1: Differences in abiotic H2 loss (in %) among laboratories.


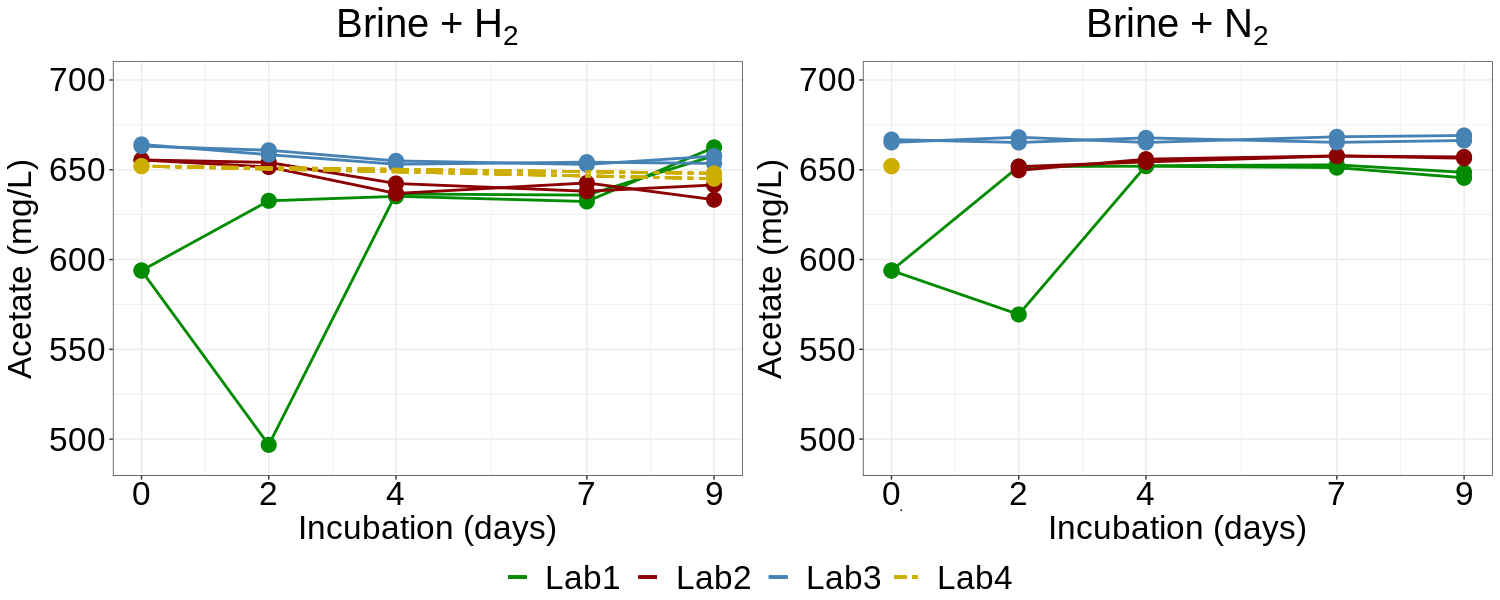
Supplementary Figure S2: Acetate concentration (mg/L) in brine samples incubated with hydrogen (left) and nitrogen (right) during the experiment in respective laboratories. Lab 4 performed only start and end-point measurements due to technical limitations.


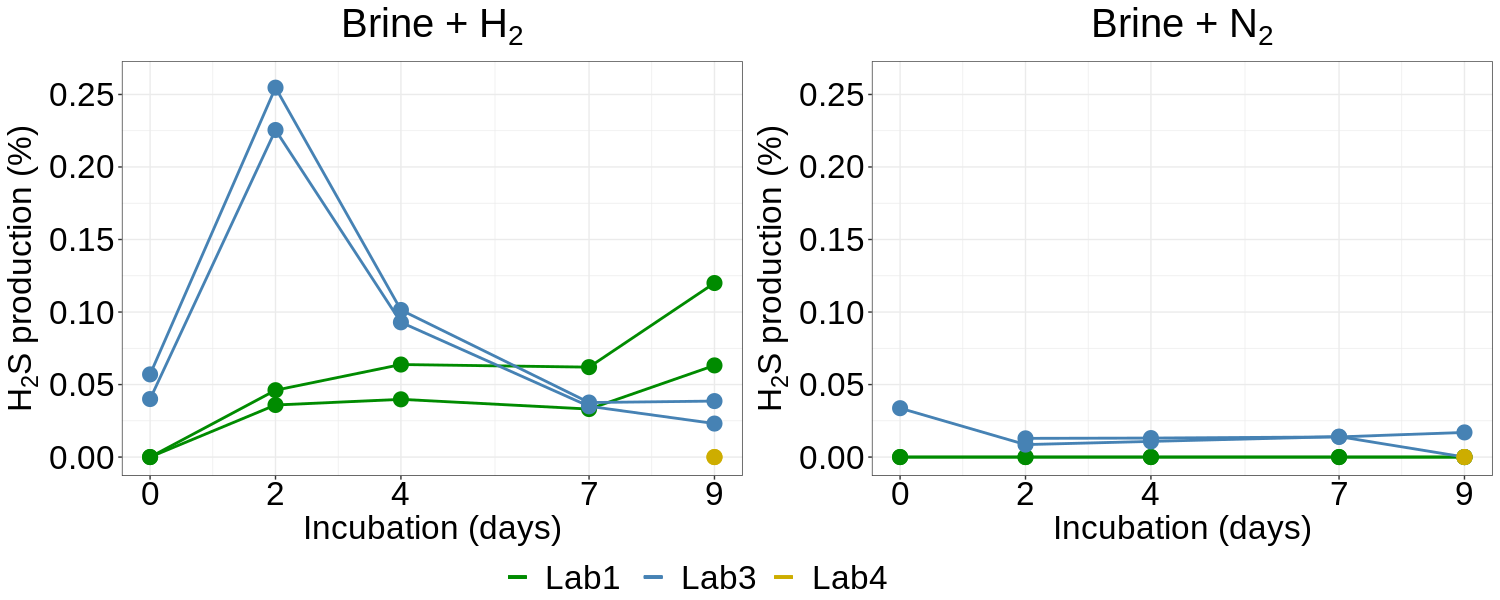

Supplementary Figure S3: Production of H2S (in %) in brine samples incubated with hydrogen (left) and nitrogen (right) during the experiment in respective laboratories. Lab 2 did not perform any H₂S measurements, while Lab 4 conducted only end-point measurements due to technical limitations.


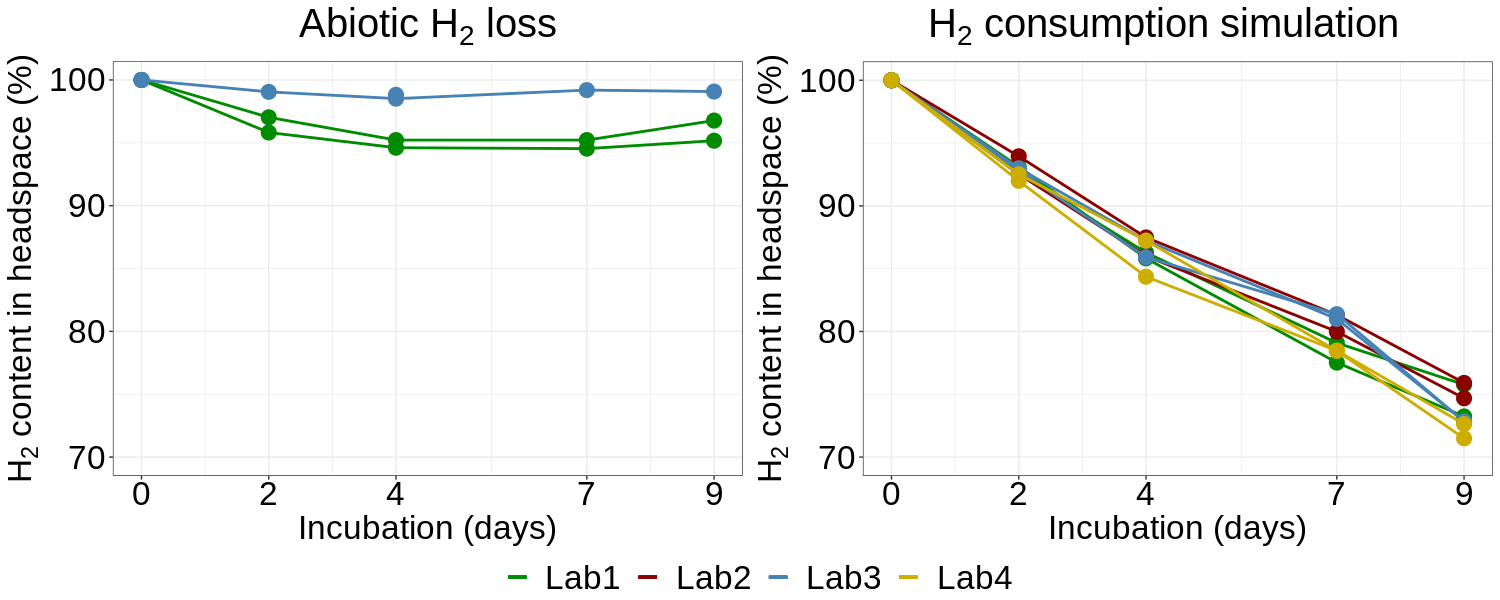


Supplementary Figure S4: Left: Abiotic H_2_ loss in Lab 1 and Lab 4 after protocol optimization. Right: Simulation of microbial H_2_ consumption (in %) demonstrating consistency in hydrogen loss detection among the labs.
